# Effects of Various Processing Methods on the Nutritional Quality and Carcinogenic Substances of Bactrian Camel (*Camelus bactrianus*) Meat

**DOI:** 10.3390/foods11203276

**Published:** 2022-10-20

**Authors:** Rendalai Si, Dandan Wu, Qin Na, Jing He, Li Yi, Liang Ming, Fucheng Guo, Rimutu Ji

**Affiliations:** 1 Key Laboratory of Dairy Biotechnology and Bioengineering, Ministry of Education, College of Food Science and Engineering, Inner Mongolia Agricultural University, Hohhot 010018, China; 2 School of Life Science and Technology, Inner Mongolia University of Science and Technology, Baotou 014010, China; 3 Inner Mongolia Institute of Camel Research, Alxa 737300, China

**Keywords:** Bactrian camel meat, processing method, nutrition, carcinogenic substance

## Abstract

Bactrian camel (*Camelus bactrianus*) meat, as a product of national geographical indication, is mainly produced in the northwest regions of China. This study systematically evaluated the edible quality, nutritional quality, and carcinogenic substances of Bactrian camel meat using different heating times in four thermal processing methods (steaming, boiling, frying, and microwaving). Compared with the control group (uncooked), the thermal processing of meat demonstrated lower redness and moisture content; higher shear force values and protein, fat, and ash contents; and sharply increased the levels of amino acids and fatty acids. The moisture content of the fried and microwave-treated meat was significantly lower than that of the steamed and boiled meat (*p* < 0.05). Steamed meat was higher in protein but had a lower fat content than the other three processing methods (*p* < 0.05). Compared with frying and microwaving, meat from steaming and boiling showed higher levels of essential amino acids and lower shear force values. However, the smoke generated during frying led to the formation of large amounts of polycyclic aromatic hydrocarbons (PAHs) and nitrites, and the levels of these substances increased with heating time. In addition, with the extension of the heating time, the shear force of the meat also increased gradually (*p* < 0.05). In summary, steaming and boiling were proven to be suitable processing methods for preserving better nutritional values while delivering less carcinogenic risk. With our results, we have established a nutritional database for Bactrian camel meat, providing a reference for selecting a suitable thermal processing method.

## 1. Introduction

Currently, with the popularization of dietary health perspectives, the importance of meat as a source of high-quality protein and micronutrients is fully recognized [1]. Camel meat is considered suitable for human consumption because of its high protein, low fat, and low cholesterol characteristics [2,3,4]. Camel meat is also receiving increased interest on a worldwide scale due to its high functional properties and nutritional values [5]. Moreover, it is a good source of inorganic minerals, essential amino acids, vitamins, biologically active compounds, linoleic acid metabolites, and essential fatty acids, such as n-6/n-3 fatty acids [6]; it is also believed to possess therapeutic functions in diseases such as hyperacidity, cardiovascular disease, hypertension, pneumonia, sciatica, and respiratory diseases [7]. Compared to other meat, such as beef and lamb, camel meat is particularly lean and is thus more suitable for the nutritional demands of consumers [8,9].

Bactrian camel meat, as a product of national geographical indication, is mainly produced in the northwest regions of China [9]. In China, the camel meat product type is unitary; its product yield is low, its market scale is small, and it cannot meet the increasing demands of customers. Therefore, the healthy profile of camel meat products in China is a promising future prospect as a tool to enhance the value of this functional meat.

At present, to extend meat product shelf life, to enhance mouthfeel and flavor, to improve digestibility, and to inactivate pathogenic microorganisms, heat treatment (cooking) is essential before the meat is consumed [10]. Heat treatment can induce a variety of changes in meat, such as chemical and physical properties, and nutritional values [11,12,13,14]. Previous studies have investigated the effects of roasting, grilling, and stewing on the quality of Spanish light lamb, observing marked alterations in vitamin and fat content; meanwhile, in cured goat leg meat, maturing mainly caused changes in protein and fat content [15,16]. Changes in the safety and nutritional indicators of Heng goat leg meat from China were detected under seven common processing methods. The results showed that heat treatment in an environment exposed to smoke led to the formation of large amounts of nitrites and polycyclic aromatic hydrocarbons (PAHs), which increased the nitrite content of fried meat and barbecued meat by 5.8 and 3.3 times, respectively, and increased the PAH levels by 3.8 and 6 times, respectively [17]. Meat products may be contaminated by carcinogenic polycyclic aromatic hydrocarbons (PAHs) during smoking [18]. In addition, it has also been reported that with an increase in heating time, the moisture content of meat decreases, but the protein content and the degree of oxidation of polyunsaturated fatty acids increase. At the same time, the thiobarbituric acid value (TBA) of pork increased with the extension of the heating time [19]. Therefore, using different processing methods and choosing the most suitable heating time are key to ensuring the nutritional quality and safety of meat. However, there has been little information on the effect of processing methods on the nutritional values of camel meat and, in particular, on the development of meat products of Bactrian camel. It is well established that obtaining such information is vital if cooking can induce changes in muscle components and hence might influence the meat’s nutritive value.

This research aimed to provide a scientific basis for improving the nutritional value of camel meat products and a theoretical basis for consumer home cooking with regard to the nutritional quality of camel meat and the level of carcinogenic substances.

## 2. Materials and Methods

### 2.1. Animals and Meat Samples

In this experiment, 11250 g of *semitendinosus* muscle samples (STs, at the thickest part of the hind legs) of Alxa Bactrian camels (n = 3, 3 years old, male, body weight 424 ± 12 kg) was selected at random from the Lifa Agriculture and Animal Husbandry Industry Co., Ltd., Bayanhot, Inner Mongolia, China, vacuum packaged (Packaging Sealing Machine, QT-124, Shanghai, China) in polyvinylidene chloride bags (Zhenzhun Biotechnology Co., Ltd., Shanghai, China; thickness 70 μm, O^2^ diffusion rates 0.05 cm^3^·m^2^·d^−1^), transported to the meat laboratory, and preserved at −20 °C. Connective tissue and fat were then removed, and the meat samples (75 samples, each sample weighing 150 g) were cut into pieces of 3 cm (length) × 2 cm (width) × 1 cm (height).

### 2.2. Thermal Processing Methods

Seventy-five meat samples from the Bactrian camel were randomly allocated to five processing treatments (untreated raw meat was the control group). For each specific processing group, 15 meat samples were prepared at random.

The samples to be tested were thawed at 4 °C for 24 h before testing and were analyzed in the same laboratory. The specific processing methods of the four heat treatment groups were as follows:

Steaming: The meat steaks were trimmed to the same size and shape, and all samples were prepared. The steamer was preheated for five minutes, and all meat cuts were placed in the steamer for different heating times (20, 25, 30, 35, and 40 min). After processing, the samples were cooled to 22–25 °C, vacuum packaged, sealed, frozen, and prepared for further testing. Three replications were prepared for each heating treatment.

Boiling: Water was brought to the boil in a pot, and the pieces of meat were added to the pot and boiled for 5 different times (20, 25, 30, 35, and 40 min). After that, the meat samples were cooled to 22–25 °C, vacuum frozen, sealed, and prepared for further testing. Three replications were prepared for each heating time period.

Frying: The meat cuts were trimmed to the same size and shape and were prepared for processing. Edible oil (50 mL) was added to a pan and heated, and then, the meat pieces were added and fried for certain periods (3, 4, 5, 6, and 7 min), while being turned over every 1 min. After treatment, the meat cuts were cooled to 22–25 °C, vacuum packaged, sealed, frozen, and prepared for further testing. Three replications were prepared for each heating time treatment.

Microwaving: The meat steaks were trimmed to the same size and shape for further processing. The meat was placed in a tray and heated in a microwave at 750 W for various times (3, 4, 5, 6, and 7 min), while being turned every 1 min. After heating, the meat was cooled to 22–25 °C, vacuum packaged, sealed, and frozen. Three replications were prepared for each individual time period.

The thermal processing groups are named in Table 1.

### 2.3. Determination of Parameters Affecting Meat Edible Quality

Parameters affecting meat quality, including pH, Warner–Bratzler shear force, and color values (a* for redness, L* for lightness, and b* for yellowness), were determined.

The Chinese recommended standard (GB5009.237 2016) method was used to determine the ultimate pH value of the camel meat as follows [20]: 10.0 g of the sample was made into minced meat, added to 90 mL of water, and shaken for 30 min in a water bath at 22 °C, and the pH of the filtrate was determined using a pH meter equipped with a glass electrode (FE28 Benchtop pH Meter, Mettler Toledo, Leicester, UK). Each sample was tested in triplicate. 

A colorimeter (Konica Minolta, CR-400, Tokyo, Japan) was used to determine the color of the meat, recording the values of L*, a*, and b*. The determination was based on previously described methods [21]. The chromameter was set to the L*, a*, or b* color space and illuminant D65; an observer angle of 2°; and an aperture size of 5.0 mm with a closed cone. The chromameter was calibrated using a standardized white tile before measurement. 

The shear force of the meat samples was assessed following the procedure described by previous research [6,21]. The shear force was obtained using a TA-XT ExpressC Texture Analyzer (Weixun Super Technology Co., Ltd., Beijing, China) fitted with a Warner–Bratzler attachment. The test probe was an HDP/BSW probe, and the measurement parameters were a pre-test rate of 2.0 mm/s, a mid-test rate of 2.0 mm/s, a post-test rate of 15.0 mm/s, and a press distance of 15.0 mm. Each sample was tested in triplicate and the average value of the measurement was reported.

### 2.4. Nutritional Quality Determination Methods

The proximate chemical composition of the meat samples was determined according to the procedure described by Liu et al. [20] and Dai et al. [22], with some modifications. The samples to be tested were thawed at 4 °C for 24 h before testing and were analyzed in the same laboratory. The ash content was determined according to the method of total ash in food in GB 5009.4-2016, and ashing samples were tested in a muffle furnace at 500 °C for 24 h. The Kjeldahl method in GB5009.5-2016 was used to measure the protein content. The GB 5009.3-2016 direct drying method was used for moisture content determination. The Soxlet extraction method in GB5009.6-2016 was used to measure the fat content. 

The fatty acids were detected based on Chinese recommended standard GB5009.168-2016 using previously described methods [9], with some modifications. The samples were extracted using a mixed solution of chloroform and methanol (*v*:*v*, 2:1) to obtain the fat. Next, each camel fat sample (10 mg) was weighed, transferred into a centrifuge tube, and hydrolyzed into fatty acids using 2 mL of 0.1 M methanolic sodium hydroxide solution. Then, 2 mL of a boron fluoride–methanol solution was added to obtain the fatty acids. Finally, 2 mL of n-hexane was added to extract the fatty acid methyl esters. A gas chromatographic system (SCION 456-GC, STS Shanghai Analytical Instrument Co., Ltd., Shanghai, China) combined with a flame ionization detector (FID) and an Agilent J&WCP-Sil88FAME capillary column (100 × 0.25 mm × 0.20 µm; Agilent Technology, Santa Clara, CA, USA) was used to analyze the contents of the fatty acid methyl esters. The temperatures of the injector and detector were set at 270 and 280 °C, respectively; the carrier gas was nitrogen; the split ratio was 100:1; and the injection volume was 1.0 μL. The detection conditions needed to satisfy the theoretical plate number (n) of at least 2000/m, and the resolution (R) was at least 1.25. The percentage of fatty acids was calculated using the area normalization method. Determinations were made on each of the collected muscle samples in triplicate. Triplicate determinations were made on each muscle sample collected.

fatty acid *Yi* (%) = (*A_Si_* × *F*_FAME*i*-FA*i*_)/∑*A_Si_* × *F*_FAME*i*–FA*i*_

where *Yi* represents the percentage of a certain fatty acid in the sample to the total fatty acid, %; *A_Si_* represents the peak area of each fatty acid methyl ester in the sample measurement solution; *F*_FAME*i*–FA*i*_ represents the fatty acid methyl ester *i* conversion coefficient to fatty acid; and ∑*A_Si_* represents the sum of the peak area of each fatty acid methyl ester in the sample measurement solution.

The amino acid content was measured according to GB5009.124-2016. The protein was broken up into free amino acids by acid hydrolysis at 105 °C for 22 h, separated using an AJS-01 amino acid special analytical column (C18, 3 μm, 4.6 × 150 mm; Welch Technology Co., Ltd., Shanghai, China), and derivatized using a ninhydrin solution. The derivatives were detected using a high-performance liquid chromatography system (HPLC, LC-20A, Shimadzu; fluorescence detector, RF20A, Shimadzu, Japan). The amino acid levels of the processed muscle samples are expressed as the average of three replicate tests in g/100 g, as described by Liu et al. [20].

### 2.5. Determination of Nitrites and PAHs

According to the spectrophotometric method of GB 5009.33-2016, the content of nitrites in meat samples was detected, and the results are expressed in mg/kg. According to the second method (gas chromatography–mass spectrometry) of GB 5009.265-2016, the PAH content of the camel meat samples from the four processing methods was measured for 11 kinds of PAHs, and the results are expressed in mg/kg.

### 2.6. Data Statistics and Analysis

In this experiment, Microsoft Excel 2010 (Microsoft Corp., Redmond, WA, USA) and IBM SPSS version 24.0 (IBM Corp., Armonk, NY, USA) were used for data processing and statistical analyses. The processed meat data were analyzed using the one-way analysis of variance (ANOVA) to examine the effect of heating time on the content of amino acids and fatty acids. When a significant effect (*p* ≤ 0.05) was detected, the means were compared using Duncan’s *t*-test.

A two-way analysis of variance (ANOVA) model was used to evaluate the fixed effects of the processing methods and processing time periods on the different traits (PH, lightness, redness, yellowness, and PAHs) using IBM SPSS version 24.0.

For the overall analysis, all traits were included in the model. Meat from the different heating times of four processing methods (steaming, boiling, frying, and microwaving) were analyzed using a model with fixed effects for the processing method, processing heating time, and processing method × processing heating time interaction in a two-way ANOVA, with the cooking batch as a random term across all cooking treatments. The ANOVA tables that were obtained were further analyzed to compare the means using least significant difference (LSD) procedures. Origin Lab software version 2021 (Origin Lab, Northampton, MA, USA) was used for graphing. The level of significance of the difference was *p* ≤ 0.05, and *p* > 0.05 was not significant. All data are expressed as the mean ± standard error of the mean (SEM).

## 3. Results

### 3.1. Effect of Different Thermal Processes on Camel Meat Consumption Qualities

#### 3.1.1. Effect of Different Thermal Processes on the pH Value of Camel Meat

The results of the pH measurements of the meat are shown in Table 2. There was an effect of processing method, processing time, and processing method × processing time interaction in the pH values (*p* < 0.05). After the steaming treatment, the pH value increased greatly, from 6.09 in the control group to its highest value of 6.40 after heating for 25 min. The pH value of microwave-treated camel meat was lower compared with that of the control group (*p* < 0.05). The pH values of the steaming treatment in CM2 (6.40) and CM3 (6.36) were significantly higher than those in the frying and microwaving treatment groups (*p* < 0.05). The different heating times resulted in significant differences in the pH value of the camel meat (*p* < 0.05), and the pH values of the camel meat in the steaming and boiling treatment groups were higher than those in the frying and microwaving treatment groups.

#### 3.1.2. Effect of Different Thermal Processes on the Color Difference of Camel Meat

Table 3 reports the effects of the heat processes on the color parameters of camel meat. There was an effect of the processing method and the processing method × processing time interaction on the L* values (*p* < 0.05) but no effect of the processing time (*p* > 0.05). For a*, there was an effect of the processing method and the processing time (*p* < 0.05), but a two-way processing method × processing time interaction (*p* > 0.05) was not observed. There was an effect of the processing method in b* (*p* < 0.05), but no effect of the processing time and the processing method × processing time interaction (*p* > 0.05).

Compared with the control group, the different processing treatments increased the L* and b* values of camel meat (*p* < 0.05) and significantly reduced the a* value (*p* < 0.05). The L* value after steaming for 20 min was the highest (52.82) and was significantly higher than that of the other heat processing treatment groups (*p* < 0.05); however, the L* value increased initially and then decreased as the heating time was extended. After microwave heating for 3 min, the a* value increased to 13.49 and then decreased to 6.96 after 7 min. Thus, the redness value was significantly reduced. The b* value after heat treatment was significantly higher than that of the control group (*p* < 0.05), and the influence of steaming and boiling was greater than that of frying or microwaving. These results showed that different heating times and methods had a certain effect on the color of the camel meat, and, within a certain time range, a longer heating time caused the overall color of the camel meat to become darker. If the cooking time is too long, the meat will be scorched and brown.

#### 3.1.3. Influence of Different Thermal Processes on the Shearing Force of Camel Meat

There was an effect of the processing method and processing time on the shear force values (*p* < 0.05), but a two-way processing method × processing time interaction (*p* > 0.05) was not observed (shown in Table 4). The shearing force increased from 9.74 kg in the control group to 33.35 kg in the frying group after 7 min. In the CM5 group, the shear forces of the frying and microwaving groups exceeded 30 kg and were significantly higher than those of the steaming and boiling groups (*p* < 0.05). There was no significant difference in the shearing force of camel meat between the steaming and boiling treatments (*p* > 0.05). The above results show that with the extension of the heating time, the shearing force of camel meat treated with the four processes increased significantly (*p* < 0.05). At the same time, the shearing forces of the meat treated by frying and microwaving were higher than those treated by steaming and boiling (*p* < 0.05). 

### 3.2. Effect of Different Thermal Processes on the Nutritional Quality of Camel Meat

**Figure 1:**
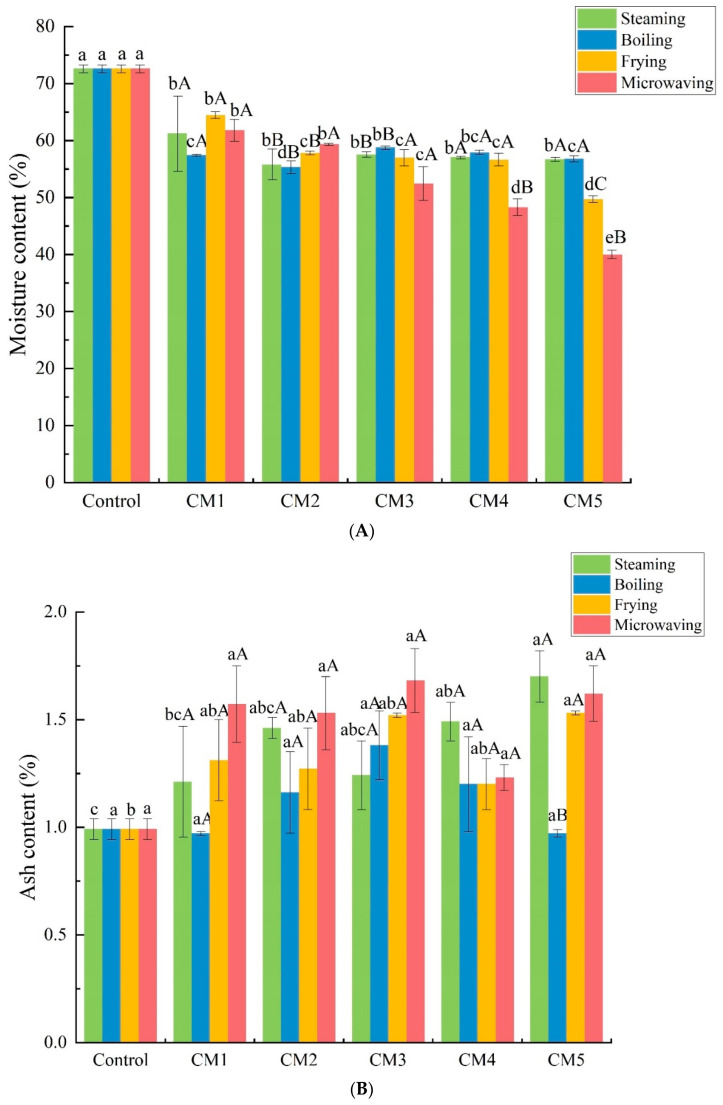

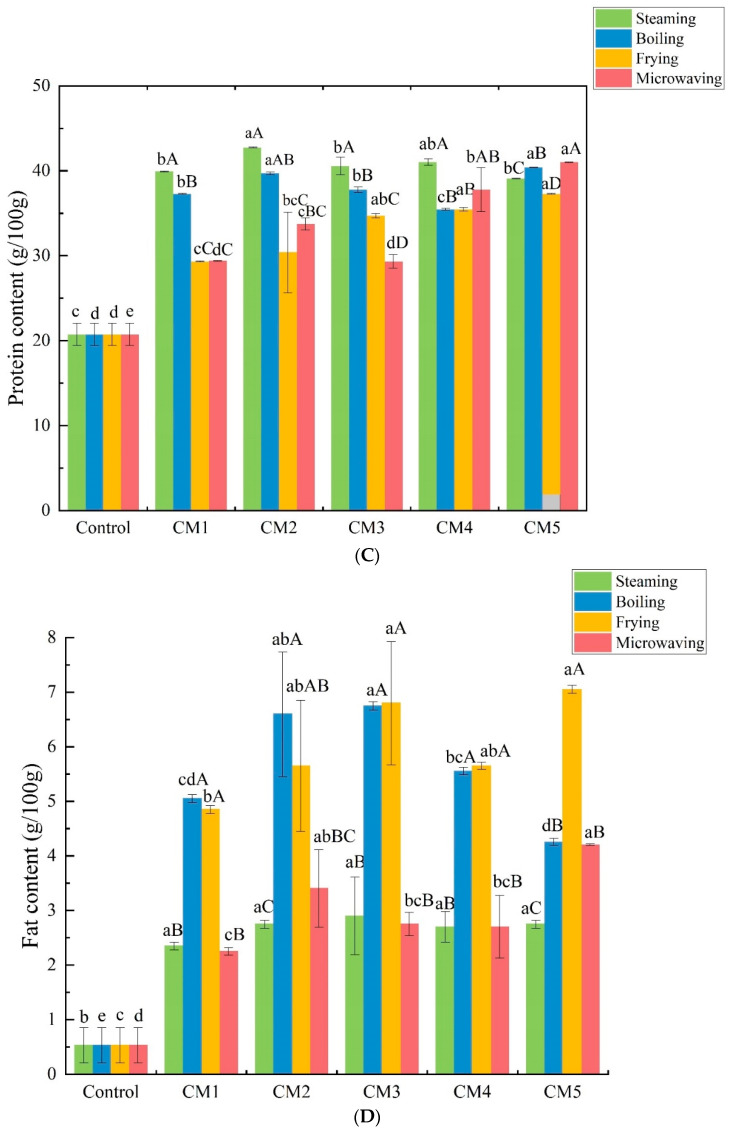
Comparison of basic nutrient compositions of camel meat after different heat processing methods. Subfigures (**A**–**D**) show the comparison graphs for moisture, ash, protein, and fat content, respectively. Different lowercase letters indicate significant differences according to the different heating times (*p* < 0.05), and different capital letters indicate significant differences according to the different heating methods (*p* < 0.05). The control group was untreated. The definitions of CM1, CM2, CM3, CM4, and CM5 are shown in Table 1.

As illustrated in Figure 1A, the moisture content of the camel meat in the control group was 72.54%, which was higher than that in the four heat treatment groups (*p* < 0.05). The water content of the camel meat was greatly reduced by the frying and microwaving treatments, whereas the water contents after steaming and boiling were both above 55%, indicating a better water holding capacity. This difference could just be because of the addition of water due to the cooking method. With increasing heating time, the ash content increased, but the difference was not significant (*p* > 0.05) (Figure 1B). The different processing methods had little effect on the ash, and there was no significant difference between the control group and the steaming, boiling, and microwaving treatment groups (*p* > 0.05). 

By comparison, the four different heat treatments increased the protein content of the meat samples (*p* < 0.05) (Figure 1C). The protein content of the meat samples was highest (42.70 g/100 g) when the meat was steamed for 25 min, and it was the lowest when the meat was fried for 3 min or microwaved for 3 min. The results show that part of the meat protein will increase with the extension of the heating time. Compared with the other treatment groups, the steamed camel meat had a higher protein content. 

Based on the results of the total fat content summarized in Figure 1D, compared with the control group, the four processing treatments significantly increased the fat content of camel meat (*p* < 0.05). The meat in the boiling and frying treatment groups had a higher fat content than that in the microwaving and steaming treatment groups. Of the four heating methods, the steaming group had the lowest fat content, 2.35–2.90 g/100 g. Meanwhile, with the extension of the heating time, the fat content of the camel meat treated by the four processes increased first and then decreased, and the difference was significant (*p* < 0.05).

### 3.3. Effect of Different Thermal Processes on Amino Acid and Fatty Acid Content of Camel Meat

#### 3.3.1. Effect of Different Thermal Processes on Amino Acid Content of Camel Meat

Amino acid composition was also analyzed to indicate the nutritional quality of the camel meat. Eight essential amino acids and eight nonessential amino acids were detected and quantified in the control and processed samples (Supplementary Table S1).

The total amino acid (TAA) content after steaming for 35 min was 39.36 g/100 g, which was significantly higher than that in the other heating periods (*p* < 0.05). The TAA, essential amino acid (EAA), and nonessential amino acid (NEAA) contents after boiling for 25 min and 40 min increased significantly compared with those in the other heating periods (*p* < 0.05). There was no significant difference in the amino acid content in the frying group (*p* > 0.05). Among them, the steamed meat not only had the highest total amino acid (TAA) content but also the richest content for all the individual essential amino acids tested, except for histidine, the highest value of which was presented in the microwaved meat. Furthermore, an increased total nonessential amino acid (NEAA) content was revealed in the meat treated with the four processing methods. Glutamic acid was shown to be the dominant nonessential amino acid in all the tested samples, followed by aspartic acid and alanine.

Compared with the other treatment times of frying, the TAA, EAA, and NEAA content were higher at 7 min of frying (*p* < 0.05). The amino acid content of the camel meat was the highest when the meat was heated by microwaving for 7 min, compared with that in the other heating periods. The amino acid content in the camel meat at 5 min of microwaving was lower, and the TAA content, EAA content, and NEAA content after 7 min of microwaving were all higher. The results also show that with the extension of the heating time, the TAA and EAA content of the camel meat treated with the four processes increased significantly (*p* < 0.05). 

#### 3.3.2. Effects of Different Thermal Processes on the Fatty Acids of Camel Meat

In the current study, the contents of 37 fatty acids, along with the levels of saturated fatty acids (SFAs), monounsaturated fatty acids (MUFAs), and polyunsaturated fatty acids (PUFAs), were analyzed in the control and processed samples to indicate the meat’s nutritional conditions after different thermal processing methods (Supplementary Table S2). Among all the detected fatty acids, myristic acid (C14:0), palmitic acid (C16:0), stearic acid (C18:0), oleic acid (C18:1n-9c), and linoleic acid (C18:2n-6c) had higher content. The fatty acids of C6:0, C8:0, C13:0, C18:3n6, C21:0, C20:3n3, C23:0, C22:2n6, C24:0, and C24:1 were not found in the control group but showed up in some of the processed meats.

The SFA content of the thermally processed meat was higher than in the control group, except for the frying group. For the steaming treatment, the MUFA content in the camel meat steamed for 30 min was higher, and the PUFA content after steaming for 40 min was higher. However, the PUFA/SFA ratio was the smallest. The PUFA content after boiling for 25 min was significantly lower compared with that in the control group (*p* < 0.05). The SFA content was 38.40% after frying for 7 min, which was significantly lower than that in the other groups (*p* < 0.05). The n-6/n-3 ratio of the frying treatment group was higher than the other groups. The PUFA content of the control group and the group treated by frying for 7 min was 17.63% and 26.04%, respectively, being significantly higher than that of the other treatment groups (*p* < 0.05). In light of these results, the trends of the total fatty acid content of the steaming and boiling treatments are similar, while the fatty acid content of the frying treatment group was higher compared with that in the control group. With the extension of the heating time, the MUFA content of the steaming group was significantly decreased (*p* < 0.05), but the PUFA content of the frying group was significantly increased (*p* < 0.05). Apart from this, among the four thermally processed groups, the fried meat demonstrated higher (*p* < 0.05) UFA and PUFA content than that of the other processed groups. The frying method gave rise to a relatively high PUFA content, with the PUFA to SFA (P/S) ratio ranging between 0.4 and 0.69, significantly higher than the P/S values detected in the other groups (0.21 to 0.32).

### 3.4. Impact of Different Thermal Processing on Carcinogenic Substances in Camel Meat 

#### 3.4.1. Effect of Different Thermal Processing on Nitrite Levels in Camel Meat

**Figure 2:**
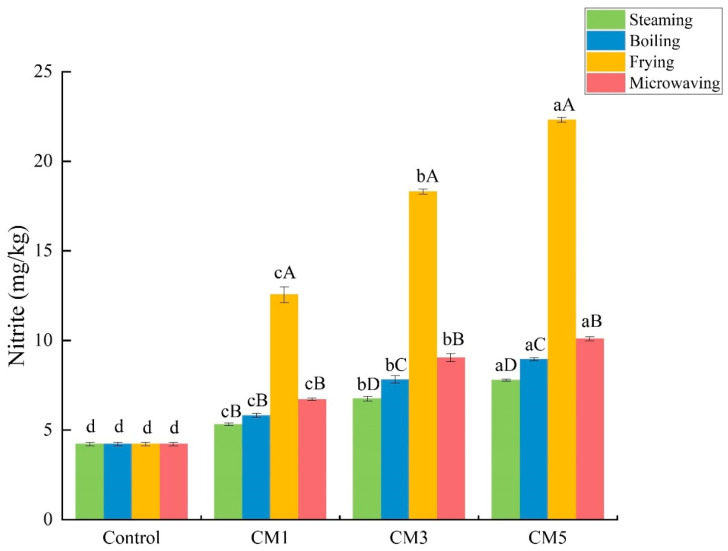
Comparison of nitrite residues in camel meat after different heat processing methods. Different lowercase letters indicate significant differences according to the different heating times (*p* < 0.05), and different capital letters indicate significant differences according to the different heating treatments (*p* < 0.05). The control group was untreated. The definitions of CM1, CM2, CM3, CM4, and CM5 are shown in Table 1.

The control group contained 4.21 mg/kg of nitrite residues, which increased to 22.29 mg/kg in the frying group in CM5. Overall, the different heat processing treatments increased the nitrite residues in the camel meat significantly compared with those in the control group (*p* < 0.05) (Figure 2). As the heating time increased, the nitrite residues increased significantly (*p* < 0.05); in CM3 and CM5, the frying treatment increased the nitrite residues from 12.55 mg/kg to 22.29 mg/kg, and there was a significant difference compared with the residues from the other groups (*p* < 0.05), in the following order: frying > microwaving > boiling > steaming. 

#### 3.4.2. Effect of Different Thermal Processing on PAHs in Camel Meat

The current investigation identified and quantified eleven PAHs in the control and processed samples (Table 5). There was an effect of the processing method in all of the PAHs (*p* < 0.05). Except for fluoranthene and benzo(a)anthracene, there was also an effect of the processing time in the PAHs (*p* < 0.05). The naphthalene, fluorene, fei, fluoranthene, and benzo (g, h, i) perylene values in the meat exhibited a pronounced influence of the processing method × processing time interaction (*p* < 0.05).

Six hazardous substances were detected after steaming for 20 min. With the extension of the heating time, the levels of multiple PAHs increased significantly (*p* < 0.05). Nine kinds of PAHs were detected after boiling and microwaving treatments. Ten kinds of PAHs were detected in the frying treatment group, and the PAH content produced after 7 min of frying was significantly higher than that of the other groups (*p* < 0.05). Among them, the levels of naphthalene, acenaphthene, phenanthrene, fluoranthene, and benzo (g, h, i) perylene after 7 min of frying were 0.205, 0.172, 0.126, 0.045, and 0.104 mg/kg, respectively, all of which were significantly higher than those in the other treatment groups (*p* < 0.05). The benzo(a)pyrene content in the camel meat was 0.136 mg/kg at 5 min and 0.139 mg/kg at 7 min of frying, whereas benzo(a)pyrene was not detected in the other treatment groups. Thus, compared with the other three groups, more types of PAHs were detected in the frying treatment group, and the PAH content was higher with the extension of the heating time. Therefore, steaming for 20 min was better for meat processing.

## 4. Discussion

The pH value is a major factor indicating meat quality and has a certain impact on the tenderness of meat. The pH value of the differently processed camel meat ranged from 5.82 to 6.40, which was consistent with the research results of Mitra et al. [23]. During the heating process, protein is denatured and cleaved, after which the basic residues of amino acids are exposed, resulting in a reduction in acidic groups and an increase in pH values [24]. Differences in the pH between different processing methods may be due to changes in the hydrolysis degree of fats to fatty acids during heating, and the hydrolysis degree of fatty acids may be due to differences in heating intensity [19].

The color of meat products is often one of the important factors that affect consumers’ purchasing intentions [9,14]. In the current study, the thermal processing methods increased the L* and b* values of camel meat and dramatically reduced a* values in contrast to that of the control, which was in good agreement with previous findings [14,17]. Compared with frying and microwave treatment, camel meat from steaming and boiling exhibited higher L* and b* values but showed lower a* values. It can be caused by multiple factors involved in water loss, the denaturation of myoglobin, and the enhanced light reflection from a meat surface due to the accumulation of moisture exuded from the muscles [16,25]. Steamed and boiled camel meat showed higher L* values, which may be due to more water exuded from the muscle inside and accumulation on the muscle surface during the heating process. The accumulation of water further enhanced the reflection ability of light and led to an increase in brightness [25]. As is well known, the observed muscle color is mainly derived from myoglobin and the changes in color mainly depend on the concentration and existence state of myoglobin [19]. Simultaneously, the concentration and oxidation state of myoglobin mainly affect the a* value (redness) of muscles [16]. A longer heating time caused the dramatic decreasing a* values of steamed and boiled meat in this paper. These results indicated higher myoglobin degradation as cooking time increased. This loss of redness with increasing heating time was in accordance with the results obtained by previous studies [16,25,26]. In addition, there was a significant increase in b* values in all thermal processing methods, especially boiling and steaming treatment in the current research. This result was consistent with that of Zhuang and Savage [26]. This was most likely due to the formation of metmyoglobin and further heat-denaturation of myoglobin, which gave rise to a brownish color [11]. With the extension of heating time, the surface of camel meat turned brown and showed a higher b* value as a consequence of Maillard reaction and caramelization reaction, which had also been reported by other authors [11,16]. Research also reported that, in a low pH environment, the conversion rate of myoglobin and oxygenated myoglobin to metmyoglobin will be increased, which can affect the color (L*, a* and b*) of the meat [27]. 

The shearing force can directly reflect the tenderness of meat products, which is an objective indicator commonly used at home and abroad to reflect meat tenderness. The smaller the shearing force, the better the tenderness of the meat [28]. The shearing force value is mainly related to the water content in the meat product. The results showed that the thermally processed meats possessed obviously higher values of shearing force compared to that of the uncooked meats. Meanwhile, the longer the heating time, the greater the water loss, which increases the shearing force of the sample. This could mainly be explained by the enhanced muscle structure caused by protein denaturation and moisture loss during thermal treatments [1]. There have also been reports to support this result, with high processing temperatures and long processing times resulting in the formation of crusts on the meat surface, affecting meat quality characteristics [11]. Processing methods such as frying and microwaving can easily form eschar on the surface of the meat, while wet processing methods such as steaming and boiling make the meat surface moist, resulting in another factor for the shearing force differences between the differently processed meats. 

As the most abundant chemical component, water usually accounts for more than 70% of the total weight of raw camel meat [2,28] and is directly related to the quality of the meat, such as color, tenderness, juiciness, and flavor [12,16]. In contrast to the control group, the moisture content of the meat in the four thermally processed treatment groups decreased dramatically, ranging from 41.3% in the microwaved meat to 64.2% in the fried meat. Due to the wet processing method, the steamed and boiled meat maintained the expected relatively high moisture content, while the microwaving method produced samples with a relatively low moisture content. This phenomenon was consistent with results reported in other literature where steaming and boiling methods produced higher moisture content in meat than the dry-air processing methods [29,30]. Simultaneously, according to the current results, the moisture content of the thermally processed camel meat decreased with the extension of the heating time. It is possible that the heating treatment causes damage to the nonpolar amino acids and the surrounding protective semi-crystalline water structure, leading to the formation of sulfhydryl bonds, which would reduce water retention and increase the loss of water [31]. 

In the current study, the protein content was significantly increased in the four thermally processed samples compared to that of the control. Protein is easy to denature when meat experiences water loss, and the extent of protein denaturation primarily depends on the conditions of the thermal processing, including heating temperature, time, and meat composition [14,32]. Meanwhile, the steamed and boiled camel meat had a higher protein content than the fried and microwaved camel meat. With the frying and microwaving cooking methods, the denaturation speed of the protein was accelerated as the result of rapid and high-temperature heating, leading to the exposure of the hydrophobic groups and the aggregation of precipitation, as well as a relatively low protein content [14,33]. In addition, with the extension of the heating time, the protein content of the steaming, boiling, frying, and microwaving treatment groups ranged from 29.25 to 42.70 g/100 g, and the increasing rate of protein in the microwave-treated group was the most rapid.

The total fat content results indicated that the four processing treatments dramatically increased the fat content of the camel meat in the current research. Meanwhile, with the extension of the heating time, the fat content of the camel meat treated by the thermal processes increased first and then decreased. This is because the water loss rate in the meat was greater than the fat loss rate during the initial heating process, resulting in a relatively high fat content. When the water loss reaches a certain level, when the thermal degradation rate of fat is higher than the water loss rate, the percentage of fat begins to decrease, which leads to the fat content increasing first and then decreasing, which is similar to the result of Broncano [34]. At the same time, the frying method had the greatest effect on the fat content of the camel meat, which could mainly be due to the addition of rapeseed oil in the frying process [35]. However, the steaming method had a minimal impact on the fat content. Meat lower in fat is more acceptable as part of a healthy and balanced diet [36]. From this point of view, steaming should be recommended rather than frying.

The content and type of amino acids in meat and meat products affect the protein content of the meat [37]. The EAA content is a key indicator to evaluate protein levels [21]. The present study indicated that the camel meat products in the steamed and boiled treatment groups had higher EAA content than those in the frying and microwaving groups. This shows that various cooking methods have obvious effects on the amino acid content of meat, and the results are consistent with the conclusion of a previous study [38]. Leucine and lysine were the two most abundant essential amino acids in all samples, while tryptophan was the only missing essential amino acid. This result is consistent with that reported by Yang et al. [19]. The presence of lysine was found to give added nutritional value to goat meat because it is a limiting amino acid in cereal-based diets, especially in developing countries [39]. Therefore, steaming is the best method for acquiring the highest amino acid content and the most digestible protein in diets. The amino acid content obtained by frying and microwaving methods is relatively low.

Fatty acid composition is one of the most important indicators in measuring the nutritional value of meat, which is closely related to meat flavor and human health [40]. It has been reported that different processing methods, due to their different heat sources, will affect the degree of fat oxidation and the composition ratio of fatty acids in meat, as well as ultimately affecting the edible quality and nutritional value of meat products [19]. In the current research, the four processing treatment groups showed significantly increased SFA levels but decreased UFA content (except for frying). The fried meat group demonstrated the highest PUFA content and P/S values than those of the other processed groups. This could be caused by the permeation of the rapeseed oil added during the meat processing, as C18:2 is the main constituent of edible oil (rapeseed oil), and the higher the amount of rapeseed oil used in processing, the higher the P/S ratio observed in processed meats. With the extension of the heating time, the MUFA content of the steaming group was significantly decreased, but the PUFA content of the frying group was significantly increased. Studies have shown that MUFAs are beneficial to health. It is recommended to replace some SFAs with MUFAs in the diet to increase MUFA intake and to help balance the blood lipid content [41]. The PUFA/SFA content in beef and mutton is about 0.1, and the value recommended by nutritionists is 0.4 [35]. However, the PUFA/SFA value of processed camel meat is between 0.21 and 0.69, which is clearly higher than that of the beef and mutton in this study. Unsaturated fatty acids and polyunsaturated fatty acids in lipids can effectively reduce serum cholesterol, in particular, n-6/n-3 and conjugated linoleic acid, which are beneficial to human health [42]. The ideal value range of n-6/n-3, as recommended by the Chinese Nutrition Association, is 4–6. The results of this study showed that the value of n-6/n-3 from the ST muscle ranged between 4.37 and 12.57. From this point of view, for consumers who pay more attention to health, boiled meat from camels is the best choice.

Eating meat products containing excessive amounts of nitrite in the diet can cause poisoning [43]. The results showed that the nitrite content in the camel meat samples of the frying treatment group was significantly higher than that of the other treatment groups. This was similar to the results of Jing et al. [44], who reported that cooking methods such as pickling, frying, steaming, smoking, and roasting have a significant effect on the formation of nitrosamines in meat. The content of nitrosamines in fried poultry and livestock meat was higher than that of other cooking methods [44]. They also found that the longer the cooking time, the more nitrosamines appeared in ham, and the higher the cooking temperature, the faster the nitrosamine value increased. 

PAHs have been found to have direct impacts on the disease burden caused by multiple cancers in the human population, and large amounts of PAHs are primarily generated during food thermal treatment [45]. Currently, 16 PAHs are listed as priority pollutants by the U.S. Environmental Protection Agency. Among them, benzo[a]pyrene and dibenzo [a.b] anthracene are classified as strong carcinogens by the International Agency for Research on Cancer. Naphthalene, benzo[a]anthracene, benzo[b]fluoranthene, benzo[k]fluoranthene, benzo[a] pyrene, indeno [1,2,3,-c,d] pyrene, diphenyl, and [a,h] anthracene are included in “China’s environmental priority pollutant blacklist” [46]. The formation of PAHs in meat products mostly comes from fat coking and cracking, protein pyrolysis, and incomplete sugar burning. The results showed that with the extension of the heating time, the four kinds of thermally processed camel meats produced certain levels of carcinogenic substances. Among them, the benzo[a]pyrene content detected in the camel meat processed by frying for 5 and 7 min was the highest, at 0.136 and 0.139 mg/kg, respectively, with their potential carcinogenicity being greater at these levels. Therefore, considering the types and content of PAHs, cooking techniques such as steaming, boiling, and microwaving are recommended, rather than frying. From the perspective of healthy consumption, meat thermal processing conducted in water should be recommended instead of in smoke exposure circumstances.

## 5. Conclusions

This is the first systematic study of the effects of four methods of thermal processing (microwaving, frying, boiling, and steaming) on the nutrition and safety quality of meat from the Bactrian camel. Our findings indicated that the protein, water, total amino acid, and monounsaturated fatty acid contents in the steaming treatment group were higher, and the fat content was lower. In addition, in terms of human health, frying is not recommended in the daily diet. The longer the frying time, the more carcinogenic substances will be produced. In summary, steaming and boiling processes were observed to be the most suitable methods in preserving the nutritional value of camel meat while minimizing the risk of carcinogenesis. These methods are the most suitable thermal processing methods recognized by the public.

## Figures and Tables

**Table 1:** Nomenclature for the five different thermal processing times.

Name of Sample Group	Thermal Processing Time
CM1	Steaming 20 min, Boiling 20 min, Frying 3 min, Microwaving 3 min
CM2	Steaming 25 min, Boiling 25 min, Frying 4 min, Microwaving 4 min
CM3	Steaming 30 min, Boiling 30 min, Frying 5 min, Microwaving 5 min
CM4	Steaming 35 min, Boiling 35 min, Frying 6 min, Microwaving 6 min
CM5	Steaming 40 min, Boiling 40 min, Frying 7 min, Microwaving 7 min

Group CM1 represents a group of camel meat samples that were steamed for 20 min, boiled for 20 min, fried for 3 min, and microwaved for 3 min. Groups CM2–CM5 represent the same as CM1 but with different heating times.

**Table 2:** Comparison of pH values of camel meat after different heat processing methods.

Processing Time	Processing Method			
	Steaming	Boiling	Frying	Microwaving
Control	6.09 ± 0.02 ^d^	6.09 ± 0.02 ^d^	6.09 ± 0.02 ^bc^	6.09 ± 0.02 ^a^
CM1	6.24 ± 0.00 ^bA^	6.21 ± 0.03 ^cA^	6.14 ± 0.02 ^aB^	5.82 ± 0.03 ^cC^
CM2	6.40 ± 0.01 ^aA^	6.14 ± 0.01 ^dB^	6.08 ± 0.03 ^bcBC^	6.03 ± 0.04 ^abC^
CM3	6.36 ± 0.01 ^aA^	6.28 ± 0.04 ^bA^	6.09 ± 0.13 ^bB^	5.95 ± 0.10 ^bB^
CM4	6.12 ± 0.01 ^dB^	6.37 ± 0.02 ^aA^	5.97 ± 0.04 ^cC^	5.95 ± 0.01 ^bC^
CM5	6.17 ± 0.01 ^cB^	6.30 ± 0.03 ^abA^	6.16 ± 0.04 ^bB^	6.02 ± 0.02 ^abC^
Two-way ANOVA (*p*-values)	*p*_processing method_ < 0.05	*p*_processing time_ < 0.05	*p*_method×time_ < 0.05	

Different lowercase letters in the same column indicate significant differences according to the different processing times (*p* < 0.05), and different capital letters in the same row indicate significant differences according to the different processing methods (*p* < 0.05). *p*_processing method_, *p*_processing time_, and *p*_method×time_ are the effects of the processing method, processing time, and the method × time interaction on the pH values of the meat, respectively. The control group was untreated. The definitions of CM1, CM2, CM3, CM4, and CM5 are shown in Table 1.

**Table 3:** Comparison of color difference of camel meat after different heat processing methods.

Color Parameters	Processing Time	Processing Method			
		Steaming	Boiling	Frying	Microwaving
L* (lightness)	Control	35.7 ± 0.0 ^e^	35.7 ± 0.1 ^f^	35.7 ± 0.1 ^e^	35.7 ± 0.1 ^d^
	CM1	52.8 ± 0.0 ^aA^	48.9 ± 0.3 ^cC^	50.8 ± 0.2 ^aB^	44.8 ± 0.1 ^cD^
	CM2	47.4 ± 0.1 ^cC^	50.8 ± 0.1 ^aA^	48.2 ± 0.1 ^bB^	47.6 ± 0.2 ^bC^
	CM3	50.7 ± 0.2 ^bA^	45.4 ± 0.1 ^eC^	42.5 ± 0.0 ^dD^	49.1 ± 0.0 ^aB^
	CM4	44.7 ± 0.1 ^dC^	47.6 ± 0.0 ^dB^	50.7 ± 0.1 ^aA^	47.40 ± 0.2 ^bB^
	CM5	47.7 ± 0.1 ^cB^	49.8 ± 0.1 ^bA^	46.4 ± 0.1 ^cC^	45.2 ± 0.5 ^cD^
Two-way ANOVA (*p*-values)		*p*_processing method_ < 0.05	*p*_processing time_ = 0.44	*p*_method×time_ < 0.05	
a* (redness)	Control	19.9 ± 0.1 ^a^	19.9 ± 0.1 ^a^	19.9 ± 0.1 ^a^	19.9 ± 0.1 ^a^
	CM1	7.7 ± 0.0 ^eD^	7.9 ± 0.1 ^cC^	11.9 ± 0.1 ^bA^	9.4 ± 0.1 ^dB^
	CM2	8.4 ± 0.1 ^bC^	8.2 ± 0.0 ^bD^	11.9 ± 0.0 ^bB^	13.5 ± 0.1 ^bA^
	CM3	7.9 ± 0.1 ^dC^	7.9 ± 0.1 ^cC^	9.0 ± 0.0 ^dB^	9.7 ± 0.1 ^cA^
	CM4	8.1 ± 0.1 ^cC^	7.6 ± 0.1 ^dD^	10.1 ± 0.0 ^cA^	8.4 ± 0.0 ^eB^
	CM5	7.8 ± 0.0 ^deB^	7.2 ± 0.1 ^eC^	8.9 ± 0.0 ^eA^	6.9 ± 0.1 ^fD^
Two-way ANOVA (*p*-values)		*p*_processing method_ < 0.05	*p*_processing time_ < 0.05	*p*_method×time_ = 0.32	
b* (yellowness)	Control	10.4 ± 0.0 ^e^	10.4 ± 0.0 ^d^	10.4 ± 0.0 ^e^	10.4 ± 0.0 ^e^
	CM1	13.8 ± 0.0 ^bcA^	13.9 ± 0.1 ^bA^	12.4 ± 0.1 ^cC^	12.8 ± 0.1 ^cB^
	CM2	13.9 ± 0.1 ^bA^	14.1 ± 0.0 ^bA^	12.8 ± 0.0 ^bB^	12.9 ± 0.1 ^cB^
	CM3	14.6 ± 0.1 ^aA^	12.7 ± 0.1 ^cB^	12.2 ± 0.1 ^dD^	12.4 ± 0.1 ^dC^
	CM4	13.4 ± 0.1 ^dB^	14.1 ± 0.3 ^bA^	13.2 ± 0.0 ^aB^	13.4 ± 0.0 ^bB^
	CM5	13.6 ± 0.1 ^cB^	14.4 ± 0.1 ^aA^	12.8 ± 0.1 ^bC^	13.7 ± 0.0 ^aB^
Two-way ANOVA (*p*-values)		*p*_processing method_ < 0.05	*p*_processing time_ = 0.09	*p*_method×time_ = 0.76	

Different lowercase letters in the same column indicate significant differences according to the different processing times (*p* < 0.05), and different capital letters in the same row indicate significant differences according to the different processing methods (*p* < 0.05). *p*_processing method_, *p*_processing time_, and *p*_method×time_ are the effects of the processing method, processing time, and the method × time interaction on the color values of the meat, respectively. The control group was untreated. The definitions of CM1, CM2, CM3, CM4, and CM5 are shown in Table 1.

**Table 4:** Comparison of the shearing force (kg) of camel meat after different heat processing methods.

Processing Time	Processing Method			
	Steaming	Boiling	Frying	Microwaving
Control	9.7 ± 3.5 ^c^	9.7 ± 6.1 ^c^	9.7 ± 6.1 ^c^	9.7 ± 6.1 ^c^
CM1	12.9 ± 0.1 ^bcA^	12.8 ± 1.6 ^bcA^	19.6 ± 9.3 ^bcA^	15.5 ± 4.1 ^bcA^
CM2	17.3 ± 0.3 ^abA^	15.2 ± 5.9 ^bcA^	20.5 ± 6.6 ^abcA^	19.1 ± 0.4 ^bA^
CM3	19.0 ± 1.4 ^aAB^	17.9 ± 1.2 ^abB^	20.4 ± 2.5 ^abcAB^	22.9 ± 1.1 ^bA^
CM4	21.2 ± 0.5 ^aBC^	19.3 ± 1.6 ^abC^	27.0 ± 6.1 ^abAB^	28.9 ± 2.4 ^aA^
CM5	21.5 ± 0.4 ^aB^	25.4 ± 3.5 ^aB^	33.4 ± 0.1 ^aA^	31.7 ± 6.1 ^aA^
Two-way ANOVA (*p*-values)	*p*_processing method_ < 0.05	*p*_processing time_ < 0.05	*p*_method×time_ = 0.90	

Different lowercase letters in the same column indicate significant differences according to the different processing s (*p* < 0.05), and different capital letters in the same row indicate significant differences according to the different processing methods (*p* < 0.05). *p*_processing method_, *p*_processing time_, and *p*_method×time_ are the effects of the processing method, processing time, and the method × time interaction on the shear force values of the meat, respectively. The control group was untreated. The definitions of CM1, CM2, CM3, CM4, and CM5 are shown in Table 1.

**Table 5:** Comparison of polycyclic aromatic hydrocarbons (mg/kg) in camel meat after different heat processing methods.

Measurements	Processing Time	Processing Method			
		Steaming	Boiling	Frying	Microwaving
Naphthalene	CM1	0.049 ± 0.009 ^cB^	0.046 ± 0.003 ^cB^	0.047 ± 0.002 ^cB^	0.104 ± 0.006 ^cA^
	CM3	0.143 ± 0.005 ^bC^	0.115 ± 0.005 ^bD^	0.165 ± 0.006 ^bB^	0.183 ± 0.004 ^aA^
	CM5	0.175 ± 0.006 ^aB^	0.165 ± 0.005 ^aB^	0.205 ± 0.004 ^aA^	0.125 ± 0.003 ^bC^
	Effect	*p*_processing method_ < 0.05	*p*_processing time_ < 0.05	*p*_method×time_ < 0.05	
Acenaphthylene	CM1	-	-	-	-
	CM3	0.046 ± 0.004 ^a^	0.044 ± 0.004 ^a^	-	-
	CM5	0.046 ± 0.004 ^a^	0.046 ± 0.005 ^a^	-	-
	Effect				
Acenaphthene	CM1	0.058 ± 0.002 ^bB^	0.073 ± 0.004 ^aAB^	0.128 ± 0.002 ^aA^	-
	CM3	0.146 ± 0.003 ^aA^	0.098 ± 0.002 ^aA^	0.149 ± 0.001 ^aA^	0.117 ± 0.003 ^A^
	CM5	0.146 ± 0.004 ^aAB^	0.071 ± 0.099 ^aB^	0.172 ± 0.007 ^aA^	0.139 ± 0.001 ^AB^
	Effect	*p*_processing method_ < 0.05	*p*_processing time_ < 0.05	*p*_method×time_ = 0.08	
Fluorene	CM1	-	0.059 ± 0.001 ^bB^	0.076 ± 0.004 ^bA^	0.084 ± 0.007 ^abA^
	CM3	0.086 ± 0.005 ^abAB^	0.070 ± 0.001 ^abB^	0.083 ± 0.007 ^aAB^	0.093 ± 0.007 ^aA^
	CM5	0.094 ± 0.006 ^aA^	0.073 ± 0.005 ^aC^	0.087 ± 0.003 ^aAB^	0.080 ± 0.007 ^bB^
	Effect	*p*_processing method_ < 0.05	*p*_processing time_ < 0.05	*p*_method×time_ < 0.05	
Fei	CM1	0.070 ± 0.014 ^bB^	0.070 ± 0.014 ^bB^	0.105 ± 0.002 ^bcA^	0.106 ± 0.004 ^bA^
	CM3	0.122 ± 0.007 ^aA^	0.096 ± 0.005 ^aB^	0.118 ± 0.002 ^abA^	0.121 ± 0.007 ^abA^
	CM5	0.131 ± 0.004 ^aA^	0.097 ± 0.003 ^aB^	0.126 ± 0.005 ^aA^	0.133 ± 0.005 ^aA^
	Effect	*p*_processing method_ < 0.05	*p*_processing time_ < 0.05	*p*_method×time_ < 0.05	
Anthracene	CM1	0.024 ± 0.003 ^bB^	0.024 ± 0.001 ^aB^	0.028 ± 0.001 ^bA^	0.030 ± 0.001 ^bA^
	CM3	0.029 ± 0.001 ^aB^	0.026 ± 0.001 ^aB^	0.029 ± 0.001 ^bB^	0.034 ± 0.002 ^aA^
	CM5	0.029 ± 0.001 ^aB^	0.027 ± 0.001 ^aB^	0.034 ± 0.002 ^aA^	0.031 ± 0.001 ^abAB^
	Effect	*p*_processing method_ < 0.05	*p*_processing time_ < 0.05	*p*_method×time_ = 0.07	
Fluoranthene	CM1	-	-	0.040 ± 0.001 ^a^	-
	CM3	0.037 ± 0.001 ^A^	0.036 ± 0.001 ^A^	0.042 ± 0.005 ^aA^	-
	CM5	0.038 ± 0.001 ^B^	0.037 ± 0.004 ^B^	0.045 ± 0.006 ^aA^	0.042 ± 0.002 ^AB^
	Effect	*p*_processing method_ < 0.05	*p*_processing time_ = 0.07	*p*_method×time_ < 0.05	
Benzo(a)anthracene	CM1	0.063 ± 0.003 ^dAB^	0.060 ± 0.001 ^aB^	0.071 ± 0.001 ^bA^	0.068 ± 0.001 ^bAB^
	CM3	0.065 ± 0.006 ^dB^	0.062 ± 0.001 ^aB^	0.084 ± 0.003 ^aA^	0.068 ± 0.001 ^bB^
	CM5	0.075 ± 0.004 ^aB^	0.065 ± 0.006 ^aC^	0.086 ± 0.005 ^aA^	0.082 ± 0.007 ^aAB^
	Effect	*p*_processing method_ = 0.36	*p*_processing time_ = 0.34	*p*_method×time_ = 0.48	
Bend	CM1	0.019 ± 0.001 ^aB^	0.020 ± 0.001 ^aB^	0.035 ± 0.001 ^bA^	0.023 ± 0.001 ^aB^
	CM3	0.024 ± 0.003 ^aB^	0.025 ± 0.002 ^aB^	0.038 ± 0.001 ^bA^	0.026 ± 0.003 ^aB^
	CM5	0.026 ± 0.005 ^aB^	0.026 ± 0.004 ^aB^	0.048 ± 0.001 ^aA^	0.027 ± 0.004 ^aB^
	Effect	*p*_processing method_ < 0.05	*p*_processing time_ < 0.05	*p*_method×time_ = 0.19	
Benzo(a)pyrene	CM1	-	-	-	
	CM3	-	-	0.136 ± 0.003	-
	CM5	-	-	0.139 ± 0.002	-
	Effect				
Benzo (g, h, i) perylene	CM1	-	-	-	-
	CM3	0.065 ± 0.001 ^B^	0.049 ± 0.001 ^B^	0.082 ± 0.007 ^A^	-
	CM5	0.066 ± 0.001 ^B^	0.058 ± 0.001 ^C^	0.104 ± 0.004 ^A^	0.049 ± 0.001 ^D^
	Effect	*p*_processing method_ < 0.05	*p*_processing time_ < 0.05	*p*_method×time_ < 0.05	

Different lowercase letters in the same column indicate significant differences according to the different processing times (*p* < 0.05), and different capital letters in the same row indicate significant differences according to the different processing methods (*p* < 0.05). - stands for undetected. *p*_processing method_, *p*_processing time_, and *p*_method×time_ are the effects of the processing method, processing time, and the method × time interaction on the PAH values of the meat, respectively. The control group was untreated. The definitions of CM1, CM3, and CM5 are shown in Table 1.

## Data Availability

Data are contained within the article.

## Supplementary Materials

The following supporting information can be downloaded at https://www.mdpi.com/article/10.3390/foods11203276/s1, Table S1: Comparison of amino acid content (g/100 g) of camel meat after different thermal processing methods and processing times; Table S2: Comparison of fatty acid content (%) of camel meat after different thermal processing methods and processing times.
