# Supplementary material for: Effects of Various Processing Methods on the Nutritional Quality and Carcinogenic Substances of Bactrian Camel (Camelus bactrianus) Meat
Source: Foods. 2022 Oct 20;11(20):3276. doi: 10.3390/foods11203276 (PMC9602032; doi:10.3390/foods11203276)
Supplement: Supplementary file 1 [file foods-11-03276-s001.zip › foods-1914992-supplementary.pdf]

foods MDPI

Supplementary Table S1. Comparison of amino acid content (g/100 g) of camel meat after different thermal processing methods and processing times.

Processing method	Control	Steaming					Control	Boiling				
Processing time	Untreated	S-20min	S-25min	S-30min	S-35min	S-40min	Untreated	B-20min	B-25min	B-30min	B-35min	B-40min
amino acids												
LYS*	1.84±0.12^{d}	3.41±0.01^{bc}	3.57±0.15^{ab}	3.51±0.04^{b}	3.76±0.07^{a}	3.25±0.04^{c}	1.84±0.12^{d}	3.46±0.01^{b}	3.68±0.01^{a}	3.35±0.13^{bc}	3.16±0.05^{c}	3.74±0.02^{a}
PHE*	0.84±0.04^{b}	1.45±0.01^{a}	1.51±0.08^{a}	1.49±0.01^{a}	1.49±0.10^{a}	1.44±0.04^{a}	0.84±0.04^{d}	1.57±0.01^{ab}	1.68±0.03^{a}	1.32±0.14^{c}	1.42±0.07^{bc}	1.59±0.01^{a}
MET*	0.51±0.05^{d}	1.08±0.01^{bc}	1.13±0.03^{ab}	1.13±0.03^{ab}	1.20±0.02^{a}	1.04±0.01^{c}	0.51±0.05^{d}	1.10±0.01^{ab}	1.16±0.01^{a}	1.07±0.04^{b}	0.99±0.01^{c}	1.13±0.01^{ab}
ILE*	0.97±0.08^{d}	1.82±0.01^{bc}	1.92±0.07^{ab}	1.91±0.03^{ab}	2.02±0.07^{a}	1.76±0.01^{c}	0.97±0.08^{c}	1.82±0.01^{b}	1.94±0.01^{a}	1.79±0.04^{b}	1.72±0.01^{b}	1.99±0.01^{a}
LEU*	1.74±0.14^{c}	3.22±0.04^{b}	3.39±0.13^{ab}	3.33±0.07^{ab}	3.57±0.13^{a}	3.14±0.03^{b}	1.74±0.14^{c}	3.25±0.03^{b}	3.48±0.01^{a}	3.21±0.07^{b}	3.11±0.01^{b}	3.53±0.04^{a}
THR*	0.94±0.07^{d}	1.71±0.01^{bc}	1.80±0.07^{ab}	1.79±0.05^{abc}	1.91±0.05^{a}	1.67±0.01^{c}	0.94±0.07^{e}	1.76±0.02^{bc}	1.83±0.01^{ab}	1.69±0.06^{cd}	1.61±0.01^{d}	1.87±0.02^{a}
VAL*	1.02±0.08^{d}	1.95±0.01^{bc}	2.03±0.08^{ab}	2.02±0.04^{ab}	2.15±0.07^{a}	1.84±0.03c	1.02±0.08^{c}	1.91±0.01^{b}	2.04±0.01^{a}	1.90±0.06^{b}	1.87±0.01^{b}	2.12±0.04^{a}
SER	0.84±0.06^{d}	1.44±0.2^{bc}	1.50±0.07^{ab}	1.48±0.03^{ab}	1.58±0.04^{a}	1.35±0.04^{c}	0.84±0.06^{d}	1.46±0.01^{bc}	1.54±0.01^{ab}	1.40±0.06^{c}	1.38±0.04^{c}	1.57±0.02^{a}
ASP	1.91±0.10^{d}	3.74±0.01^{bc}	3.92±0.18^{ab}	3.88±0.06^{b}	4.14±0.11^{a}	3.63±0.04^{c}	1.91±0.10^{c}	3.71±0.01^{b}	3.95±0.03^{a}	3.67±0.13^{b}	3.58±0.01^{b}	4.01±0.01^{a}
GLU	3.22±0.23^{d}	5.86±0.01^{bc}	6.20±0.28^{ab}	6.14±0.12^{ab}	6.55±0.17^{a}	5.66±0.04^{c}	3.22±0.23^{d}	5.91±0.01^{b}	6.29±0.04^{a}	5.78±0.18^{bc}	5.57±0.01^{c}	6.45±0.04^{a}
ALA	1.18±0.06^{d}	2.12±0.01^{bc}	2.20±0.08^{ab}	2.16±0.04^{bc}	2.31±0.01^{a}	2.05±0.07^{c}	1.18±0.06^{d}	2.16±0.03^{bc}	2.29±0.03^{a}	2.05±0.09^{c}	2.11±0.01^{c}	2.27±0.02^{ab}
GLY	0.98±0.09^{d}	1.61±0.01^{b}	1.71±0.07^{ab}	1.67±0.01^{b}	1.83±0.06^{a}	1.46±0.01^{c}	0.98±0.09^{d}	1.77±0.01^{b}	1.84±0.11^{ab}	1.57±0.06^{c}	1.98±0.01^{a}	1.84±0.06^{ab}
HIS*	0.99±0.02^{c}	1.36±0.01^{ab}	1.39±0.08^{a}	1.37±0.04^{a}	1.44±0.05^{a}	1.27±0.02^{b}	0.99±0.02^{c}	1.37±0.01^{a}	1.42±0.01^{a}	1.23±0.05^{b}	1.28±0.07^{b}	1.41±0.01^{a}
TYR	0.73±0.04^{d}	1.29±0.01^{bc}	1.36±0.05^{ab}	1.34±0.01^{ab}	1.40±0.04^{a}	1.24±0.01^{c}	0.73±0.04^{c}	1.39±0.01^{a}	1.46±0.03^{a}	1.26±0.08^{b}	1.17±0.02^{b}	1.40±0.01^{a}
ARG	1.31±0.08^{e}	2.38±0.02^{c}	2.52±0.08^{b}	2.47±0.03^{bc}	2.65±0.01^{a}	2.25±0.02^{d}	1.31±0.08^{c}	2.43±0.01^{b}	2.56±0.03^{a}	2.35±0.08^{b}	2.36±0.01^{b}	2.63±0.01^{a}
PRO	0.75±0.15^{b}	1.28±0.01^{a}	1.33±0.12^{a}	1.24±0.06^{a}	1.39±0.01^{a}	1.24±0.01^{a}	0.75±0.15^{c}	1.54±0.01^{a}	1.51±0.08^{a}	1.21±0.08^{b}	1.42±0.01^{ab}	1.43±0.01^{a}
TAA	19.78±1.01^{d}	35.69±0.01^{bc}	37.44±1.63^{ab}	36.90±0.54^{b}	39.36±0.89^{a}	34.28±0.01^{c}	19.78±1.01^{d}	36.54±0.02^{b}	38.66±0.18^{a}	34.83±1.38^{bc}	34.67±0.03^{c}	38.87±0.02^{a}
EAA	7.85±0.55^{d}	14.62±0.01^{bc}	15.34±0.62^{ab}	15.17±0.27^{abc}	16.09±0.52^{a}	14.14±0.01^{c}	7.85±0.55^{d}	14.86±0.01^{b}	15.81±0.02^{a}	14.32±0.55^{bc}	13.85±0.02^{c}	15.89±0.02^{a}
NEAA	11.92±0.51^{d}	21.07±0.02^{bc}	22.11±1.01^{ab}	21.73±0.27^{b}	23.28±0.37^{a}	20.14±0.02^{c}	11.92±0.51^{d}	21.68±0.02^{b}	22.85±0.16^{a}	20.51±0.83^{bc}	20.82±0.03^{c}	22.98±0.01^{a}
EAA/TAA	39.69±1.13^{a}	40.96±0.02^{a}	40.97±0.13^{a}	41.11±0.13^{a}	40.87±0.39^{a}	41.25±0.01^{a}	39.69±1.13^{a}	40.67±0.01^{a}	40.89±0.13^{a}	41.12±0.04^{a}	39.95±0.03^{a}	40.88±0.02^{a}
EAA/NEAA	65.84±3.08^{a}	69.39±0.01^{a}	69.39±0.39^{a}	69.81±0.37^{a}	69.10±1.13^{a}	70.21±0.01^{a}	65.84±3.08^{a}	68.54±0.02^{a}	69.17±0.38^{a}	69.84±0.13^{a}	66.52±0.02^{a}	69.15±0.03^{a}

Supplementary Table S1 continued.

	Control	Frying					Control	Microwaving				
	Untreated	F-3min	F-4min	F-5min	F-6min	F-7min	Untreated	M-3min	M-4min	M-5min	M-6min	M-7min
LYS*	1.84±0.12^{c}	2.74±0.04^{b}	2.77±0.41^{b}	2.97±0.04^{ab}	3.11±0.21^{ab}	3.37±0.03^{a}	1.84±0.12^{d}	2.58±0.02^{c}	2.88±0.34^{c}	2.56±0.05^{c}	3.37±0.24^{b}	3.88±0.01^{a}
PHE*	0.84±0.04^{b}	1.14±0.04^{ab}	1.12±0.29^{ab}	1.23±0.01^{a}	1.37±0.23^{a}	1.41±0.01^{a}	0.84±0.04^{d}	1.02±0.03^{cd}	1.18±0.20^{c}	1.06±0.03^{c}	1.49±0.08^{b}	1.72±0.03^{a}
MET*	0.51±0.05^{b}	0.84±0.01^{a}	0.83±0.17^{a}	0.87±0.08^{a}	0.98±0.11^{a}	1.02±0.03^{a}	0.51±0.05^{e}	0.83±0.03^{cd}	0.92±0.06^{c}	0.77±0.03^{d}	1.05±0.04^{b}	1.28±0.07^{a}
ILE*	0.97±0.08^{c}	1.46±0.04^{b}	1.51±0.24^{b}	1.62±0.01^{b}	1.68±0.07^{ab}	1.88±0.01^{a}	0.97±0.08^{d}	1.45±0.07^{c}	1.49±0.08^{c}	1.36±0.02^{c}	1.77±0.11^{b}	2.05±0.02^{a}
LEU*	1.74±0.14^{c}	2.55±0.01^{b}	2.67±0.40^{b}	2.87±0.01^{b}	2.98±0.19^{ab}	3.34±0.03^{a}	1.74±0.14^{d}	2.47±0.02^{c}	2.71±0.25^{c}	2.41±0.01^{c}	3.12±0.20^{b}	3.64±0.03^{a}
THR*	0.94±0.07^{d}	1.35±0.02^{c}	1.44±0.23^{bc}	1.55±0.01^{abc}	1.64±0.10^{ab}	1.77±0.02^{a}	0.94±0.07^{d}	1.33±0.01^{c}	1.47±0.17^{c}	1.29±0.01^{c}	1.67±0.08^{b}	1.96±0.03^{a}
VAL*	1.02±0.08^{c}	1.53±0.03^{b}	1.57±0.23^{b}	1.68±0.01^{b}	1.78±0.13^{ab}	1.97±0.04^{a}	1.02±0.08^{e}	1.45±0.01^{cd}	1.62±0.14^{c}	1.43±0.01^{d}	1.84±0.11^{b}	2.12±0.01^{a}
SER	0.84±0.06^{d}	1.16±0.01^{c}	1.20±0.18^{bc}	1.32±0.01^{bc}	1.37±0.07^{ab}	1.54±0.04^{a}	0.84±0.06^{d}	1.12±0.02^{c}	1.22±0.12^{c}	1.08±0.01^{c}	1.39±0.08^{b}	1.63±0.03^{a}
ASP	1.91±0.10^{d}	2.87±0.03^{c}	3.01±0.46^{bc}	3.23±0.01^{bc}	3.42±0.24^{ab}	3.72±0.02^{a}	1.91±0.10^{e}	2.84±0.06^{cd}	3.14±0.28^{c}	2.74±0.04^{d}	3.56±0.19^{b}	4.12±0.01^{a}
GLU	3.22±0.23^{d}	4.61±0.01^{c}	4.85±0.78^{bc}	5.21±0.03^{abc}	5.53±0.37^{ab}	6.02±0.02^{a}	3.22±0.23^{e}	4.56±0.04^{cd}	5.09±0.46^{c}	4.38±0.11^{d}	5.76±0.37^{b}	6.66±0.01^{a}
ALA	1.18±0.06^{c}	1.72±0.02^{b}	1.74±0.25^{b}	1.90±0.01^{b}	1.85±0.06^{b}	2.23±0.04^{a}	1.18±0.06^{e}	1.63±0.03^{cd}	1.80±0.16^{c}	1.57±0.01^{d}	2.05±0.11^{b}	2.34±0.01^{a}
GLY	0.98±0.09^{d}	1.34±0.01^{c}	1.26±0.11^{bc}	1.52±0.04^{b}	1.50±0.08^{b}	1.85±0.06^{a}	0.98±0.09^{d}	1.25±0.07^{c}	1.41±0.02^{b}	1.17±0.01^{c}	1.54±0.08^{b}	1.74±0.01^{a}
HIS*	0.99±0.02^{b}	1.23±0.01^{ab}	1.23±0.16^{ab}	1.25±0.03^{ab}	1.51±0.29^{a}	1.45±0.01^{a}	0.99±0.02^{d}	1.15±0.07^{cd}	1.37±0.19^{bc}	1.27±0.04^{bc}	1.45±0.11^{ab}	1.64±0.03^{a}
TYR	0.73±0.04^{c}	1.02±0.02^{b}	1.03±0.16^{b}	1.08±0.01^{ab}	1.19±0.14^{ab}	1.25±0.01^{a}	0.73±0.04^{d}	0.98±0.02^{c}	1.11±0.17^{bc}	0.99±0.02^{c}	1.28±0.08^{b}	1.48±0.04^{a}
ARG	1.31±0.08^{c}	1.94±0.01^{b}	1.97±0.29^{b}	2.13±0.01^{b}	2.17±0.11^{b}	2.48±0.03^{a}	1.31±0.08^{d}	1.84±0.03^{c}	2.04±0.19^{bc}	1.77±0.02^{c}	2.23±0.21^{b}	2.68±0.01^{a}
PRO	0.75±0.15^{c}	1.06±0.01^{b}	1.07±0.13^{b}	1.21±0.04^{ab}	1.11±0.02^{b}	1.37±0.01^{a}	0.75±0.15^{d}	1.00±0.01^{c}	1.12±0.06^{bc}	0.97±0.04^{c}	1.29±0.10^{ab}	1.46±0.03^{a}
TAA	19.78±1.01^{c}	28.50±1.02^{b}	29.34±4.49^{b}	31.63±0.12^{b}	33.17±2.28^{ab}	36.55±0.01^{a}	19.78±1.01^{e}	27.38±0.02^{cd}	30.54±2.86^{c}	26.78±0.14^{d}	34.83±2.21^{b}	40.23±0.01^{a}
EAA	7.85±0.55^{d}	11.49±0.03^{c}	11.90±1.97^{bc}	12.78±0.04^{abc}	13.78±1.40^{ab}	14.75±0.01^{a}	7.85±0.55^{d}	11.04±0.03^{c}	12.26±1.26^{c}	10.87±0.01^{c}	14.30±0.86^{b}	16.54±0.03^{a}
NEAA	11.92±0.51^{d}	17.01±0.03^{c}	17.34±2.52^{bc}	18.85±0.16^{abc}	19.64±1.23^{ab}	21.80±0.01^{a}	11.92±0.51^{e}	16.34±0.01^{cd}	18.28±1.61^{c}	15.92±0.13^{d}	20.53±1.34^{b}	23.69±0.01^{a}
EAA/TAA	39.69±1.13^{a}	40.32±0.02^{a}	40.67±0.48^{a}	40.40±0.26^{a}	40.78±0.35^{a}	40.36±0.03^{a}	39.69±1.13^{a}	40.32±0.04^{a}	40.14±0.36^{a}	40.57±0.18^{a}	41.06±0.13^{a}	41.11±0.01^{a}
EAA/NEAA	65.84±3.08^{a}	67.55±0.02^{a}	68.55±1.36^{a}	67.78±0.74^{a}	68.86±1.02^{a}	67.66±0.01^{a}	65.84±3.08^{a}	67.56±0.01^{a}	67.05±0.10^{a}	68.28±0.53^{a}	69.67±0.36^{a}	69.82±0.01^{a}

Different lowercase letters in the same row (within the same processing method) indicate significant differences according to the different heating times ($P < 0.05$).
HIS—Histidine; ILEU—isoleucine; LEU—leucine; LYS—lysine; MET—methionine; PHE—phenylalanine; THR—threonine; TYR—tyrosine; VAL—valine; GLU—Glutamic acid; PRO—Prolene; ALA—Alanine; ARG—Arginine; SER—Serine; GLY—Glycine; ASP—Aspartic acid; TAA—total amino acid; EAA—essential amino acid; NEAA—nonessential amino acid.

Supplementary Table S2. Comparison of fatty acid content (%) of camel meat after different thermal processing methods and processing times.

Processing method	Control	Steaming					Control	Boiling				
Processing time / Fatty acid	Untreated	S-20min	S-25min	S-30min	S-35min	S-40min	Untreated	B-20min	B-25min	B-30min	B-35min	B-40min
C4:0	0.03±0.06^{c}	0.12±0.01^{bc}	0.16±0.05^{b}	0.14±0.04^{b}	0.26±0.03^{a}	0.19±0.01^{ab}	0.03±0.06^{c}	0.28±0.01^{a}	0.15±0.06^{b}	0.14±0.03^{b}	0.13±0.01^{b}	0.22±0.01^{ab}
C6:0	-	0.02±0.01^{bc}	0.02±0.01^{bc}	0.04±0.01^{b}	0.07±0.03^{a}	0.04±0.01^{b}	-	0.12±0.01^{a}	0.03±0.01^{c}	0.03±0.01^{c}	0.02±0.01^{c}	0.06±0.01^{b}
C8:0	-	0.01±0.01^{b}	0.02±0.01^{ab}	0.02±0.01^{ab}	0.02±0.01^{a}	0.02±0.01^{a}	-	0.02±0.01^{a}	0.01±0.01^{b}	0.02±0.01^{a}	0.01±0.01^{b}	0.02±0.01^{a}
C10:0	0.20±0.35^{a}	0.13±0.01^{a}	0.21±0.11^{a}	0.12±0.01^{a}	0.14±0.04^{a}	0.13±0.01^{a}	0.20±0.35^{a}	0.25±0.01^{a}	0.08±0.01^{a}	0.12±0.03^{a}	0.11±0.01^{a}	0.13±0.01^{a}
C11:0	-	-	-	-	-	-	-	-	-	-	-	-
C12:0	0.27±0.06^{a}	0.31±0.01^{a}	0.35±0.01^{a}	0.34±0.04^{a}	0.30±0.02^{a}	0.30±0.01^{a}	0.27±0.06^{c}	0.60±0.01^{a}	0.35±0.03^{b}	0.40±0.01^{b}	0.37±0.01^{b}	0.30±0.01^{bc}
C13:0	-	0.02±0.01^{a}	0.03±0.01^{a}	0.03±0.01^{a}	0.02±0.01^{a}	0.02±0.01^{a}	-	0.02±0.01^{a}	0.02±0.01^{a}	0.02±0.01^{a}	0.02±0.01^{a}	0.01±0.01^{a}
C14:0	5.62±1.09^{a}	6.24±0.01^{a}	6.37±0.42^{a}	6.78±0.18^{a}	6.33±0.56^{a}	5.94±0.01^{a}	5.62±1.09^{a}	5.78±0.01^{a}	7.01±0.13^{a}	6.61±0.12^{a}	6.09±0.01^{a}	6.56±0.01^{a}
C14:1	0.18±0.07^{a}	0.21±0.01^{a}	0.22±0.01^{a}	0.25±0.01^{a}	0.21±0.01^{a}	0.20±0.01^{a}	0.18±0.07^{d}	0.22±0.01^{cd}	0.37±0.01^{a}	0.32±0.01^{ab}	0.28±0.01^{bc}	0.30±0.01^{abc}
C15:0	0.44±0.15^{a}	0.60±0.01^{a}	0.59±0.04^{a}	0.59±0.06^{a}	0.63±0.04^{a}	0.60±0.01^{a}	0.44±0.15^{a}	0.60±0.01^{a}	0.49±0.03^{a}	0.55±0.07^{a}	0.52±0.01^{a}	0.60±0.01^{a}
C15:1	-	-	-	-	-	-	-	-	0.01±0.01^{a}	0.01±0.01^{a}	0.01±0.01^{a}	-
C16:0	22.52±1.02^{a}	23.98±0.01^{a}	23.56±0.66^{a}	23.94±0.42^{a}	23.33±1.04^{a}	23.08±0.01^{a}	22.52±1.02^{b}	26.25±0.01^{a}	27.06±0.98^{a}	26.80±0.34^{a}	26.11±0.01^{a}	23.08±0.01^{a}
C16:1	3.34±0.37^{ab}	3.12±0.01^{b}	3.35±0.28^{ab}	3.77±0.16^{a}	3.53±0.24^{ab}	2.98±0.01^{b}	3.34±0.37^{d}	3.83±0.01^{c}	5.26±0.08^{a}	4.85±0.07^{ab}	4.41±0.01^{b}	4.44±0.01^{b}
C17:0	0.53±0.09^{b}	0.67±0.01^{a}	0.73±0.02^{a}	0.64±0.04^{b}	0.70±0.01^{a}	0.71±0.01^{a}	0.53±0.09^{b}	0.78±0.01^{a}	0.61±0.11^{b}	0.66±0.05^{ab}	0.60±0.01^{b}	0.71±0.01^{b}
C17:1	0.48±0.05^{b}	0.59±0.01^{a}	0.58±0.02^{a}	0.59±0.01^{a}	0.60±0.02^{a}	0.59±0.01^{a}	0.48±0.05^{a}	0.54±0.01^{a}	0.56±0.01^{a}	0.57±0.06^{a}	0.55±0.01^{a}	0.59±0.01^{a}
C18:0	13.19±1.40^{a}	14.04±0.01^{a}	13.43±0.21^{a}	13.18±0.13^{a}	13.65±0.06^{a}	14.16±0.01^{a}	13.19±1.40^{a}	11.33±0.01^{ab}	10.26±0.02^{b}	10.55±0.36^{b}	11.45±0.01^{b}	10.84±0.01^{b}
C18:1n9t	0.23±0.18^{a}	0.39±0.01^{a}	0.40±0.01^{a}	0.39±0.04^{a}	0.44±0.06^{a}	0.38±0.01^{a}	0.23±0.18^{b}	0.51±0.01^{a}	0.42±0.05^{ab}	0.42±0.01^{ab}	0.39±0.01^{ab}	0.38±0.01^{ab}
C18:1n9c	33.88±1.29^{a}	30.43±0.01^{bc}	31.45±0.37^{bc}	32.00±0.49^{ab}	29.99±1.24^{bc}	29.50±0.01^{c}	33.88±1.29^{a}	30.08±0.01^{c}	32.51±0.25^{ab}	31.27±0.11^{bc}	29.96±0.01^{c}	32.26±0.01^{ab}
C18:2n6t	-	-	-	-	-	-	-	-	0.01±0.01^{a}	0.01±0.01^{a}	0.01±0.01^{a}	-
C18:2n6c	12.04±1.44^{a}	11.77±0.01^{a}	11.42±1.20^{a}	10.32±0.96^{a}	12.56±1.86^{a}	13.37±0.01^{a}	12.04±1.44^{a}	12.71±0.01^{a}	9.57±0.79^{b}	10.46±0.71^{ab}	12.29±0.01^{a}	10.98±0.01^{ab}

C20:0	0.10±0.03^{b}	0.18±0.01^{a}	0.18±0.01^{a}	0.17±0.01^{a}	0.16±0.01^{a}	0.19±0.01^{a}	0.10±0.03^{b}	0.18±0.01^{a}	0.18±0.01^{a}	0.19±0.02^{a}	0.20±0.01^{a}	0.17±0.01^{a}
C18:3n6	-	0.08±0.01^{a}	0.08±0.01^{a}	0.07±0.01^{a}	0.05±0.06^{ab}	0.09±0.01^{a}	-	-	0.02±0.03^{b}	0.06±0.01^{a}	0.06±0.01^{a}	0.08±0.01^{a}
C20:1	0.31±0.02^{a}	0.21±0.01^{b}	0.23±0.01^{b}	0.23±0.01^{b}	0.21±0.01^{b}	0.20±0.01^{b}	0.30±0.02^{ab}	0.29±0.01^{b}	0.35±0.04^{a}	0.32±0.01^{ab}	0.31±0.01^{ab}	0.30±0.01^{ab}
C18:3n3	1.76±0.10^{a}	1.12±0.01^{bc}	1.13±1.41^{bc}	0.98±0.06^{c}	1.13±0.14^{bc}	1.27±0.01^{b}	1.76±0.10^{ab}	1.68±0.01^{bc}	1.59±0.01^{c}	1.88±0.07^{a}	1.89±0.01^{a}	1.45±0.01^{d}
C21:0	-	1.01±0.01^{a}	1.02±0.15^{a}	1.05±0.10^{a}	0.91±0.09^{a}	0.92±0.01^{a}	-	0.47±0.01^{c}	0.50±0.03^{bc}	0.51±0.04^{bc}	0.52±0.01^{b}	0.60±0.01^{a}
C20:2	0.08±0.02^{a}	0.10±0.01^{a}	0.08±0.02^{a}	0.09±0.01^{a}	0.09±0.03^{a}	0.10±0.01^{a}	0.08±0.02^{a}	0.08±0.01^{a}	0.09±0.01^{a}	0.09±0.01^{a}	0.09±0.01^{a}	0.10±0.01^{a}
C22:0	0.43±0.12^{a}	0.21±0.01^{b}	0.22±0.02^{b}	0.20±0.04^{b}	0.18±0.04^{b}	0.24±0.01^{b}	0.43±0.12^{a}	0.06±0.01^{b}	0.08±0.01^{b}	0.09±0.01^{b}	0.09±0.01^{b}	0.13±0.01^{b}
C20:3n6	0.38±0.04^{b}	0.47±0.01^{ab}	0.46±0.03^{ab}	0.44±0.07^{ab}	0.50±0.06^{a}	0.51±0.01^{a}	0.38±0.04^{a}	0.30±0.01^{bc}	0.23±0.03^{d}	0.26±0.02^{cd}	0.32±0.01^{ab}	0.34±0.01^{ab}
C22:1	0.47±0.21^{a}	0.10±0.01^{b}	0.12±0.03^{b}	0.09±0.01^{b}	0.15±0.02^{b}	0.09±0.01^{b}	0.47±0.21^{a}	0.22±0.01^{ab}	0.07±0.03^{b}	0.08±0.04^{b}	0.06±0.01^{b}	0.10±0.01^{b}
C20:3n3	-	0.04±0.01^{ab}	0.05±0.01^{a}	0.04±0.01^{ab}	0.04±0.01^{ab}	0.03±0.01c	-	0.03±0.01^{c}	0.06±0.01^{a}	0.07±0.01^{a}	0.05±0.01^{b}	0.05±0.01^{b}
C20:4	2.66±0.31^{a}	0.06±0.01^{b}	0.07±0.02^{b}	0.07±0.01^{b}	0.07±0.01^{b}	0.07±0.01^{b}	2.66±0.31^{a}	0.03±0.01^{b}	0.01±0.01^{b}	0.01±0.01^{b}	0.02±0.01^{b}	0.03±0.01^{b}
C23:0	-	2.59±0.01^{a}	2.45±0.06^{a}	2.37±0.39^{a}	2.61±0.62^{a}	2.76±0.01^{a}	-	1.99±0.01^{b}	1.56±0.11^{c}	1.85±0.22^{b}	2.22±0.01^{a}	1.98±0.01^{b}
C22:2n6	-	0.07±0.01^{a}	0.06±0.01^{a}	0.06±0.01^{a}	0.07±0.02^{a}	0.07±0.01^{a}	-	0.04±0.01^{b}	0.04±0.01^{b}	0.05±0.01^{a}	0.05±0.01^{a}	0.04±0.01^{b}
C24:0	-	0.13±0.01^{a}	0.13±0.02^{a}	0.12±0.02^{a}	0.12±0.04^{a}	0.15±0.01^{a}	-	0.08±0.01^{b}	0.05±0.01^{d}	0.05±0.01^{d}	0.06±0.01^{c}	0.13±0.01^{a}
C20:5n3	0.94±0.08^{a}	0.83±0.01^{a}	0.78±0.01^{a}	0.77±0.13^{a}	0.83±0.23^{a}	0.89±0.01^{a}	0.94±0.08^{a}	0.52±0.01^{b}	0.38±0.04^{c}	0.49±0.04^{b}	0.60±0.01^{b}	0.55±0.01^{b}
C24:1	-	0.06±0.01^{a}	0.06±0.01^{a}	0.06±0.01^{a}	0.04±0.01^{c}	0.05±0.01^{b}	-	0.04±0.01^{b}	0.03±0.01^{c}	0.03±0.01^{c}	0.03±0.01^{c}	0.06±0.01^{a}
C22:6	0.10±0.03^{a}	0.11±0.01^{a}	0.10±0.04^{a}	0.13±0.04^{a}	0.12±0.04^{a}	0.15±0.01^{a}	0.10±0.03^{a}	0.06±0.01^{ab}	0.05±0.01^{b}	0.07±0.01^{ab}	0.08±0.01^{ab}	0.08±0.01^{ab}
SFA	43.33±0.62^{b}	50.43±0.24^{a}	49.42±1.21^{a}	49.67±0.56^{a}	49.40±0.97^{a}	49.48±0.04^{a}	43.33±0.62^{b}	48.79±0.04^{a}	48.42±0.72^{a}	48.54±0.63^{a}	48.51±0.01^{a}	47.84±0.04^{a}
UFA	56.51±0.66^{a}	49.77±0.01^{b}	50.59±1.23^{b}	50.32±0.58^{b}	50.60±0.95^{b}	50.54±0.01^{b}	56.51±0.66^{a}	51.14±0.06^{b}	51.59±0.70^{b}	51.45±0.65^{b}	51.43±0.04^{b}	52.18±0.04^{b}
MUFA	38.88±1.72^{a}	35.10±0.01^{bc}	36.39±0.10^{abc}	37.36±0.71^{ab}	35.16±1.53^{bc}	33.98±0.01^{c}	38.88±1.72^{a}	35.75±0.03^{c}	39.56±0.13^{a}	37.85±0.21^{abc}	36.25±0.35^{b}	38.43±0.04^{abc}
PUFA	17.63±2.04^{a}	14.66±0.01^{ab}	14.20±1.33^{ab}	12.96±1.29^{b}	15.44±2.49^{ab}	16.55±0.01^{ab}	17.63±2.04^{a}	15.43±0.03^{ab}	12.03±0.83^{c}	13.60±0.86^{bc}	15.44±0.04^{ab}	13.68±0.02^{bc}
PUFA/SFA	0.41±0.05^{a}	0.28±0.01^{b}	0.29±0.04^{b}	0.26±0.03^{b}	0.31±0.06^{b}	0.32±0.02^{ab}	0.41±0.05^{a}	0.33±0.01^{b}	0.25±0.02^{c}	0.29±0.02^{bc}	0.33±0.01^{b}	0.28±0.01^{bc}
n-6/n-3	6.16±1.24^{a}	5.73±0.01^{a}	5.72±0.30^{a}	5.55±0.13^{a}	6.11±0.38^{a}	5.81±0.01^{a}	6.16±1.24^{a}	5.66±0.02^{ab}	4.77±0.29^{ab}	4.37±0.06^{b}	4.80±0.01^{ab}	5.30±0.01^{ab}

Supplementary Table S2 continued.

	Control	Frying					Control	Microwaving				
	Untreated	F-3min	F-4min	F-5min	F-6min	F-7min	Untreated	M-3min	M-4min	M-5min	M-6min	M-7min
C4:0	0.03±0.06^{a}	0.07±0.01^{a}	0.08±0.03^{a}	0.05±0.01^{a}	0.06±0.01^{a}	0.04±0.01^{a}	0.03±0.06^{c}	0.09±0.01^{bc}	0.12±0.01^{b}	0.10±0.01^{bc}	0.12±0.02^{b}	0.26±0.01^{a}
C6:0	-	0.21±0.01^{b}	0.27±0.01^{a}	0.13±0.02^{d}	0.17±0.01^{b}	0.14±0.01^{d}	-	0.06±0.01^{ab}	0.07±0.05^{ab}	0.04±0.01^{bc}	0.09±0.01^{a}	0.07±0.01^{ab}

C8:0	-	0.08±0.01^{ab}	0.09±0.02^{a}	0.05±0.01^{c}	0.07±0.01^{abc}	0.06±0.01^{bc}	-	0.02±0.01^{a}	0.02±0.01^{a}	0.02±0.01^{a}	0.02±0.01^{a}	-
C10:0	0.20±0.35^{a}	0.09±0.01^{a}	0.10±0.02^{a}	0.06±0.01^{a}	0.07±0.01^{a}	0.05±0.01^{a}	0.20±0.35^{a}	0.14±0.01^{a}	0.14±0.08^{a}	0.11±0.02^{a}	0.09±0.03^{a}	0.09±0.01^{a}
C11:0	-	-	0.01±0.01^{a}	0.01±0.01^{a}	-	-	-	-	-	-	-	-
C12:0	0.27±0.06^{b}	0.47±0.01^{a}	0.28±0.06^{b}	0.27±0.01^{b}	0.26±0.05^{b}	0.21±0.01^{b}	0.27±0.06^{a}	0.32±0.01^{a}	0.36±0.06^{a}	0.33±0.01^{a}	0.32±0.01^{a}	0.33±0.01^{a}
C13:0	-	0.02±0.01^{a}	0.02±0.01^{a}	0.01±0.01^{b}	0.01±0.01^{b}	0.01±0.01^{b}	-	0.02±0.01^{a}	0.02±0.01^{a}	0.02±0.01^{a}	0.01±0.01^{a}	-
C14:0	5.62±1.09^{b}	7.86±0.01^{a}	4.85±1.12^{bc}	5.38±0.42^{bc}	4.34±1.40^{bc}	3.31±0.01^{c}	5.62±1.09^{a}	6.76±0.01^{a}	5.81±0.24^{a}	6.53±0.18^{a}	5.76±0.25^{a}	6.40±0.01^{a}
C14:1	0.18±0.07^{b}	0.52±0.01^{a}	0.23±0.07^{b}	0.28±0.04^{b}	0.24±0.13^{b}	0.14±0.01^{b}	0.18±0.07^{a}	0.22±0.01^{a}	0.23±0.01^{a}	0.25±0.01^{a}	0.26±0.01^{a}	0.25±0.01^{a}
C15:0	0.44±0.15^{a}	0.47±0.01^{a}	0.45±0.01^{a}	0.41±0.03^{a}	0.37±0.04^{a}	0.32±0.01^{a}	0.44±0.15^{a}	0.59±0.01^{a}	0.60±0.10^{a}	0.60±0.03^{a}	0.55±0.02^{a}	0.60±0.01^{a}
C15:1	-	-	-	0.01±0.01^{a}	0.01±0.01^{a}	0.01±0.01^{a}	-	-	0.01±0.01^{a}	-	-	-
C16:0	22.52±1.02^{c}	26.60±0.01^{a}	25.26±0.11^{a}	24.92±0.40^{ab}	23.52±1.16^{bc}	22.53±0.01^{c}	22.52±1.02^{b}	26.89±0.01^{a}	27.06±2.21^{a}	27.18±0.32^{a}	27.35±0.38^{a}	28.09±0.01^{a}
C16:1	3.34±0.37^{bc}	5.68±0.01^{a}	3.24±0.64^{bc}	3.93±0.38^{b}	3.24±1.19^{bc}	2.40±0.01^{c}	3.34±0.37^{c}	3.68±0.01^{bc}	3.96±0.13^{ab}	4.27±0.21^{a}	4.38±0.03^{a}	4.35±0.01^{a}
C17:0	0.53±0.09^{a}	0.37±0.01^{b}	0.53±0.02^{a}	0.56±0.06^{a}	0.52±0.01^{a}	0.48±0.01^{b}	0.53±0.09^{b}	0.66±0.01^{ab}	0.70±0.16^{ab}	0.63±0.03^{ab}	0.64±0.04^{ab}	0.76±0.01^{a}
C17:1	0.48±0.05^{a}	0.45±0.01^{a}	0.42±0.05^{ab}	0.47±0.04^{a}	0.44±0.08^{ab}	0.32±0.01^{b}	0.48±0.05^{b}	0.56±0.01^{a}	0.58±0.01^{a}	0.60±0.03^{a}	0.60±0.01^{a}	0.56±0.01^{a}
C18:0	13.19±1.40^{a}	5.99±0.01^{c}	9.95±0.30^{b}	9.83±0.18^{b}	9.81±0.93^{b}	9.60±0.01^{b}	13.19±1.40^{a}	13.15±0.01^{a}	11.74±0.89^{a}	11.58±0.28^{a}	11.55±0.30^{a}	11.65±0.01^{a}
C18:1n9t	0.23±0.18^{b}	0.66±0.01^{a}	0.71±0.14^{a}	0.54±0.10^{a}	0.64±0.06^{a}	0.48±0.01^{ab}	0.23±0.18^{b}	0.45±0.01^{ab}	0.47±0.08^{ab}	0.45±0.02^{ab}	0.49±0.01^{ab}	0.54±0.14^{a}
C18:1n9c	33.88±1.29^{ab}	30.54±0.01^{b}	32.65±0.94^{ab}	34.16±0.98^{a}	32.99±2.06^{ab}	31.90±0.01^{ab}	33.88±1.29^{a}	34.22±0.01^{a}	31.72±1.34^{bc}	32.62±0.25^{ab}	33.37±0.14^{ab}	30.44±0.01^{c}
C18:2n6t	-	0.02±0.01^{a}	0.01±0.01^{b}	0.02±0.01^{a}	0.02±0.01^{a}	0.01±0.01^{b}	-	-	-	-	-	-
C18:2n6c	12.03±1.44^{c}	16.87±0.01^{bc}	17.59±2.63^{b}	15.50±2.43^{bc}	19.33±4.40^{ab}	23.59±0.01^{a}	12.04±1.44^{a}	8.45±0.01^{b}	11.13±2.68^{ab}	9.33±1.00^{ab}	10.14±0.49^{ab}	10.59±0.01^{ab}
C20:0	0.10±0.03^{c}	0.10±0.01^{c}	0.29±0.04^{b}	0.30±0.01^{b}	0.32±0.04^{ab}	0.36±0.01^{a}	0.10±0.03^{b}	0.19±0.01^{a}	0.20±0.04^{a}	0.22±0.04^{a}	0.19±0.01^{a}	0.16±0.01^{a}
C18:3n6	-	0.06±0.01^{bc}	0.05±0.01^{c}	0.06±0.01^{bc}	0.07±0.01^{ab}	0.08±0.01^{a}	-	0.06±0.01^{a}	0.05±0.03^{a}	0.06±0.01^{a}	0.05±0.01^{a}	-
C20:1	0.31±0.02^{b}	0.21±0.01^{c}	0.31±0.01^{b}	0.35±0.01^{a}	0.31±0.01^{b}	0.29±0.01^{b}	0.31±0.02^{ab}	0.26±0.01^{c}	0.30±0.02^{bc}	0.31±0.01^{ab}	0.34±0.01^{a}	0.34±0.01^{a}
C18:3n3	1.76±0.10^{ab}	1.76±0.01^{ab}	1.31±0.06^{c}	1.33±0.27^{c}	1.46±0.27^{bc}	1.93±0.01^{a}	1.76±0.10^{a}	1.10±0.01^{b}	1.42±0.57^{ab}	1.45±0.04^{ab}	1.08±0.05^{b}	1.47±0.01^{ab}
C21:0	-	0.21±0.01^{a}	0.26±0.13^{a}	0.26±0.06^{a}	0.22±0.04^{a}	0.18±0.01^{a}	-	0.66±0.01^{a}	0.53±0.06^{b}	0.55±0.07^{b}	0.39±0.07^{c}	0.48±0.01^{bc}
C20:2	0.08±0.02^{a}	-	0.03±0.01^{ab}	0.03±0.01^{ab}	0.07±0.05^{a}	0.08±0.01^{a}	0.08±0.02^{a}	0.08±0.01^{a}	0.08±0.02^{a}	0.08±0.01^{a}	0.08±0.01^{a}	0.08±0.01^{a}
C22:0	0.43±0.12^{a}	0.05±0.01^{c}	0.25±0.04^{b}	0.25±0.01^{b}	0.32±0.07^{ab}	0.36±0.01^{ab}	0.43±0.12^{a}	0.11±0.01^{b}	0.13±0.02^{b}	0.12±0.01^{b}	0.15±0.01^{b}	0.11±0.01^{b}
C20:3n6	0.38±0.04^{a}	0.08±0.01^{b}	0.09±0.01^{b}	0.09±0.01^{b}	0.13±0.06^{b}	0.11±0.01^{b}	0.38±0.04^{a}	0.19±0.01^{b}	0.27±0.16^{ab}	0.24±0.03^{ab}	0.23±0.01^{ab}	0.23±0.01^{ab}
C22:1	0.47±0.01^{a}	0.04±0.01^{b}	0.04±0.01^{b}	0.04±0.01^{b}	0.05±0.01^{b}	0.02±0.01^{b}	0.47±0.21^{a}	0.06±0.01^{b}	0.09±0.04^{b}	0.07±0.06^{b}	0.06±0.01^{b}	0.09±0.01^{b}
C20:3n3	-	0.03±0.01^{a}	0.02±0.01^{a}	0.02±0.01^{a}	0.02±0.01^{a}	0.02±0.01^{a}	-	0.03±0.01^{b}	0.05±0.03^{ab}	0.06±0.01^{ab}	0.04±0.01^{ab}	0.06±0.01^{a}

C20:4	2.66±0.31^{a}	-	0.05±0.01^{b}	0.05±0.01^{b}	0.07±0.02^{b}	0.06±0.01^{b}	2.66±0.31^{a}	0.03±0.01^{b}	0.04±0.01^{b}	0.02±0.01^{b}	0.05±0.01^{b}	0.03±0.01^{b}
C23:0	-	0.42±0.01^{a}	0.41±0.11^{a}	0.45±0.01^{a}	0.59±0.27^{a}	0.57±0.01^{a}	-	0.68±0.01^{ab}	1.53±1.39^{a}	1.59±0.06^{a}	1.18±0.11^{ab}	1.46±0.01^{a}
C22:2n6	-	-	0.01±0.01^{a}	0.01±0.01^{a}	0.02±0.01^{a}	0.01±0.01^{a}	-	0.02±0.01^{a}	0.04±0.04^{a}	0.04±0.01^{a}	0.03±0.01^{a}	-
C24:0	-	-	0.12±0.01^{c}	0.13±0.01^{bc}	0.16±0.04^{ab}	0.17±0.01^{a}	-	0.07±0.01^{b}	0.09±0.02^{b}	0.07±0.02^{b}	0.12±0.01^{a}	0.08±0.01^{b}
C20:5n3	0.94±0.08^{a}	0.10±0.01^{b}	0.09±0.04^{b}	0.09±0.01^{b}	0.12±0.06^{b}	0.12±0.01^{b}	0.94±0.08^{a}	0.15±0.01^{b}	0.43±0.47^{b}	0.44±0.01^{b}	0.26±0.03^{b}	0.34±0.01^{b}
C24:1	-	-	0.01±0.01^{b}	0.02±0.01^{a}	0.03±0.01^{a}	0.02±0.01^{a}	-	0.03±0.01^{a}	0.03±0.01^{a}	0.03±0.01^{a}	0.04±0.01^{a}	0.03±0.01^{a}
C22:6	0.10±0.03^{a}	-	-	0.01±0.01^{b}	0.01±0.01^{b}	0.01±0.01^{b}	0.10±0.03^{a}	-	0.05±0.06^{ab}	0.06±0.01^{ab}	0.03±0.01^{ab}	0.02±0.01^{b}
SFA	43.33±0.62^{a}	43.04±0.04^{a}	43.17±0.90^{a}	43.03±1.17^{a}	40.78±1.34^{b}	38.40±0.01^{c}	43.33±0.62^{b}	50.40±0.02^{a}	49.08±2.40^{a}	49.66±0.49^{a}	48.51±0.76^{a}	50.53±0.02^{a}
UFA	56.51±0.66^{c}	57.01±0.01^{c}	56.84±0.88^{c}	56.99±1.19^{c}	59.23±1.34^{b}	61.50±0.14^{a}	56.51±0.66^{a}	49.57±0.03^{b}	50.91±2.41^{b}	50.35±0.51^{b}	51.50±0.74^{b}	49.39±0.05^{b}
MUFA	38.88±1.72^{a}	38.25±1.72^{a}	37.59±1.85^{a}	39.79±1.54^{a}	37.94±3.54^{a}	35.59±0.01^{a}	38.88±1.72^{ab}	39.46±0.03^{a}	37.37±1.64^{ab}	38.58±0.45^{ab}	39.53±0.13^{a}	36.50±0.14^{b}
PUFA	17.63±2.04^{b}	18.94±0.03^{b}	19.25±2.74^{b}	17.20±2.73^{b}	21.30±4.89^{a}	26.04±0.02^{a}	17.63±2.04^{a}	10.13±0.03^{b}	13.54±4.05^{a}	11.78±0.95^{b}	11.97±0.61^{b}	12.84±0.02^{b}
PUFA/SFA	0.41±0.05^{b}	0.46±0.02^{b}	0.45±0.07^{b}	0.40±0.07^{b}	0.53±0.13^{a}	0.69±0.01^{a}	0.41±0.05^{a}	0.21±0.01^{b}	0.28±0.10^{b}	0.24±0.02^{b}	0.25±0.01^{b}	0.27±0.03^{b}
n-6/n-3	6.16±1.24^{c}	9.06±0.03^{b}	12.57±0.99^{a}	11.01±0.48^{a}	12.22±0.23^{a}	11.47±0.03^{a}	6.16±1.24^{a}	6.77±0.02^{a}	6.59±2.40^{a}	4.82±0.66^{a}	7.50±0.11^{a}	5.85±0.07^{a}

Different lowercase letters in the same row (within the same processing method) indicate significant differences according to the different heating times ($P < 0.05$).
- stands for undetected.
The unit of fatty acid content is % and the ratio unit is 1.
SFA—total saturated fatty acid; MUFA—monounsaturated fatty acid; PUFA—polyunsaturated fatty acid; UFA—total unsaturated fatty acid.
